# Insights Into the Molecular Mechanisms of T Follicular Helper-Mediated Immunity and Pathology

**DOI:** 10.3389/fimmu.2018.01884

**Published:** 2018-08-15

**Authors:** Lei Qin, Tayab C. Waseem, Anupama Sahoo, Shayahati Bieerkehazhi, Hong Zhou, Elena V. Galkina, Roza Nurieva

**Affiliations:** ^1^Department of Immunology, University of Texas MD Anderson Cancer Center, Houston, TX, United States; ^2^School of Life Science and Technology, University of Electronic Science and Technology of China, Chengdu, China; ^3^Department of Microbiology and Molecular Cell Biology, Eastern Virginia Medical School, Norfolk, VA, United States

**Keywords:** T follicular helper cells, germinal center, transcription, autoimmunity, cancer

## Abstract

T follicular helper (Tfh) cells play key role in providing help to B cells during germinal center (GC) reactions. Generation of protective antibodies against various infections is an important aspect of Tfh-mediated immune responses and the dysregulation of Tfh cell responses has been implicated in various autoimmune disorders, inflammation, and malignancy. Thus, their differentiation and maintenance must be closely regulated to ensure appropriate help to B cells. The generation and function of Tfh cells is regulated by multiple checkpoints including their early priming stage in T zones and throughout the effector stage of differentiation in GCs. Signaling pathways activated downstream of cytokine and costimulatory receptors as well as consequent activation of subset-specific transcriptional factors are essential steps for Tfh cell generation. Thus, understanding the mechanisms underlying Tfh cell-mediated immunity and pathology will bring into spotlight potential targets for novel therapies. In this review, we discuss the recent findings related to the molecular mechanisms of Tfh cell differentiation and their role in normal immune responses and antibody-mediated diseases.

## Introduction

Germinal centers (GCs) are secondary lymphoid structures within B cell follicles where B cells go through affinity maturation and class-switch recombination to generate high-affinity antibodies (1, 2). GC reactions play a critical role in the invasion of pathogens, while abnormal GC reactions are implicated in systemic autoimmune diseases, chronic inflammation, allergic responses, and B cell malignancies. The GC reaction is initiated and amplified by GC B cell and CD4^+^ T cell interactions followed by T follicular helper (Tfh) cell help to B cells which leads to the generation of long-lived serological memory (2, 3). Exaggerated expansion of Tfh cells results in excessive GC reactions, self-reactive B cell proliferation, and increased long-lived plasma cell differentiation, as well as an overproduction of high-affinity pathogenic autoantibodies (4). Understanding the development and function of Tfh cells is important for generating new vaccine strategies against pathogens as well as targeted approaches to abrogate the inappropriate activity of these cells in patients with various autoimmune diseases.

**Figure 1:**
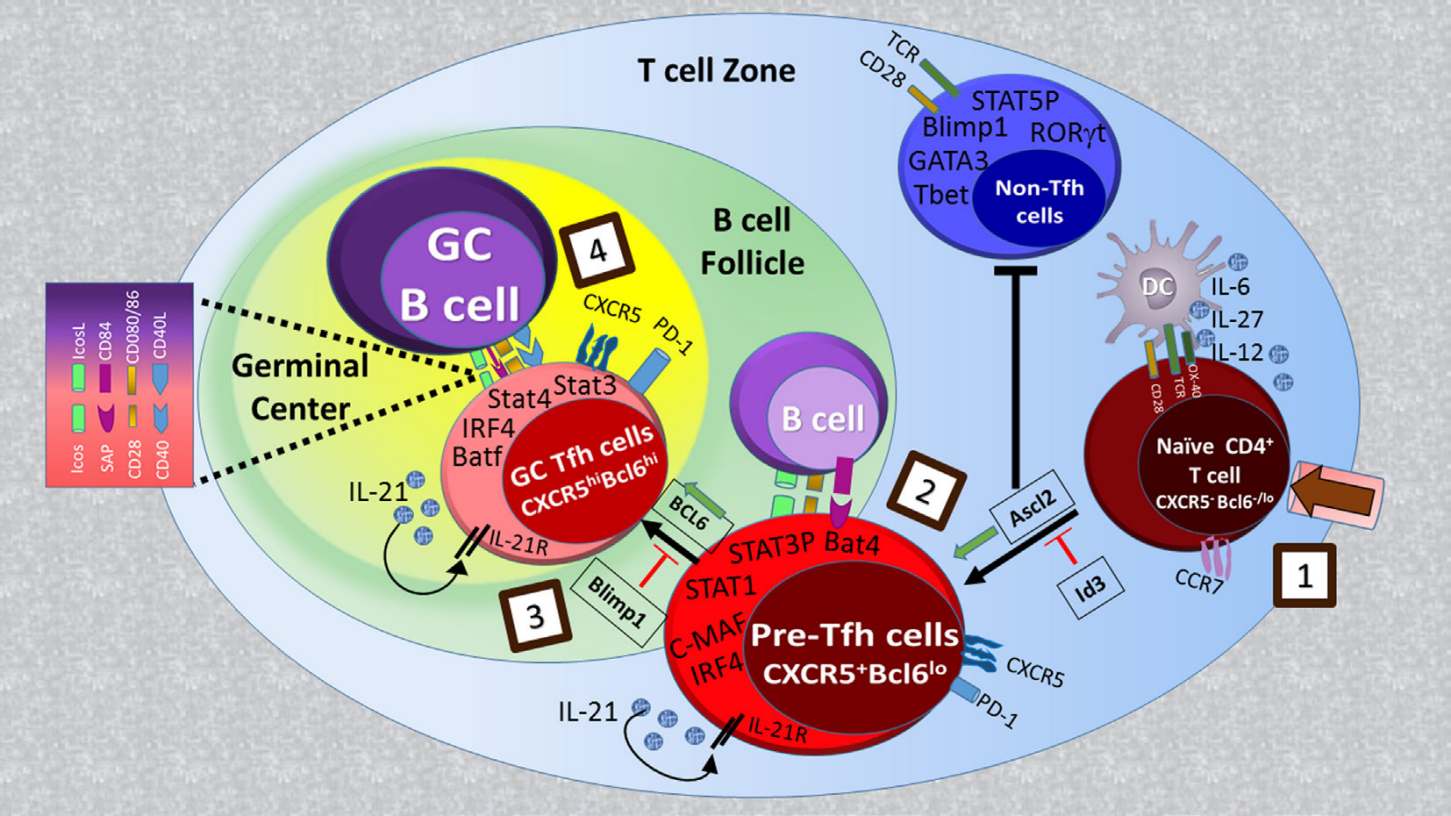
Regulatory signaling for T follicular helper (Tfh) cell development: naïve CD4^+^ T cell priming by MHC/antigen interaction on DCs (step 1) leads to the generation of CXCR5^+^Bcl6^lo^ pre-Tfh cells with increased activity of transcriptional factors such as Achaete-scute homolog 2 (Ascl2), signal transducers and activators of transcription (STAT)1, STAT3, IFN-regulatory factor 4 (IRF4), and Batf (step 2). Ascl2 expression in activated T cells orchestrates them to migrate toward B cell follicles by upregulation of CXC chemokine receptor 5 (CXCR5) expression and repressing IL-2R-Blimp1 pathway as well as Th1, Th2, and Th17 cell differentiation. Upon interaction with cognate B cells at T-B border (step 3), CXCR5^+^ T cells begin to upregulate B-cell lymphoma 6 protein (Bcl6) expression, which in cooperation with other transcription factors determines the final differentiation state of Tfh cells within germinal centers (step 4).

T follicular helper cell development occurs in a stepwise process (1, 2). Contact of naïve CD4^+^ T cells with antigen-presenting dendritic cells (DCs) within T cell follicles is the first step of commitment toward Tfh cell differentiation (Figure 1). This step of Th cell development, named as pre-Tfh is reflected by upregulation of CXC chemokine receptor 5 (CXCR5) expression as well as key genes in the Tfh pathway such as B-cell lymphoma 6 protein (Bcl6), Achaete-scute homolog 2 (Ascl2), ICOS, programmed cell death-1 (PD-1), and Batf, and the downregulation of CC chemokine receptor 7 (CCR7) expression. These changes guide the pre-Tfh cells to the T/B cell border where proper signals received from B cells trigger a further increase in the Tfh-associated gene expression pattern (Bcl6, PD-1, ICOS, and CXCR5), commitment to the functional GC Tfh cell program and subsequent GC formation. While in mice, IL-6, IL-21, and Bcl6 are essential for Tfh formation, in humans, Tfh generation relies on TGF-β, IL-12, IL-23, and Activin A signaling (5–9). In addition, Tfh cell differentiation is negatively controlled by cytokines (IL-2 and IL-7) costimulatory molecules [cytotoxic T lymphocyte antigen 4 (CTLA4) and PD-1], and transcriptional factors [signal transducers and activators of transcription (STAT)5, Blimp-1, FOXO1, Foxp1, and Krüppel-like factor 2 (Klf2)] (10–16).

In this review, we have discussed positive and negative regulation mechanisms of mouse and human Tfh differentiation including costimulation and cytokine driven signaling pathways and subsequent activation of downstream transcriptional factors. We also elaborate on the cellular requirement of other follicular T cells within GCs for Tfh cell development and GC formation. Moreover, we will review the current advances in Tfh cell biology in various disease settings.

## Tfh Cells in Mice and Humans

Several seminal discoveries made in humans and mice in the early 2000s led to identification of B follicular helper T cells that are indispensable for GC formation and B cell function (17, 18). These cells express high levels of CXCR5 and low CCR7 in both humans and mice (17–20). CXCR5 expression is indispensable for T cell migration from T zones toward CXCL13-rich B-cell follicles, where they interact with B cells for further maturation and then provide help for the generation of high-affinity antibodies and long-lived plasma cells (21–24). Thus, based on their localization and function, CXCR5^+^ CD4^+^ T cells were designated as Tfh cells.

Profiling of cytokine and gene expression patterns provided the evidence that mouse and human Tfh cells are distinct from Th1 and Th2 subsets and help B cells by delivering activating signals with CD40L and the cytokine IL-21 (3, 6, 18, 25). Moreover, these mouse and human gene-profiling experiments helped to identify key molecules including Bcl6, Ascl2, IL-21, PD-1, and ICOS which play a substantial role in Tfh cell development, migration, homeostasis, and function in both species. In 2009, three independent groups identified the essential function of the transcription factor (TF) Bcl6 for Tfh cell development and function (7, 26, 27). Since then Tfh cells have been recognized as a distinct lineage of T helper cells.

Studies in mice and humans show that Tfh cells are localized in lymphoid organs and are composed of subsets that differ in their localization, phenotype, and function (28). In mice, after priming with DCs in the T zones of secondary lymphoid organs, a fraction of naive CD4^+^ T cells acquire CXCR5, PD-1, and Bcl6 expression, downregulate CCR7, and migrate toward B cell follicles (Figure 1) (21, 29–32). These CXCR5^+^Bcl6^+^CD4^+^ T cells, called Tfh precursors (pre-Tfh), interact with antigen-presenting B cells and further differentiate to fully programmed GC Tfh cells, which provide help to B cells within GCs. GC Tfh cells can be distinguished from Tfh precursors by high expression levels of CXCR5 and PD-1 (28). Sustained expression of Bcl-6 in GC Tfh cells is essential for GC formation (7, 26, 27). The origin of Tfh cells is not restricted to naive cells, and there is some evidence suggesting that other Th subsets including Th1, Th2, Th17, and regulatory T cells (Tregs) may become Tfh cells in GCs (3). This is consistent with the heterogeneity in cytokine expression patterns among GC Tfh cells developed under different immunization protocols and by different types of infectious agents (33). In human tonsils, CXCR5^lo^ICOS^lo^ pre-Tfh cells (or extrafollicular helper T cells) express multiple Tfh molecules including CD40L, IL-21, and CXCL13 but not Bcl6 and are localized outside of GCs where they help naïve B cells to become immunoglobulin-producing cells (34). By contrast, CXCR5^hi^ICOS^hi^PD-1^hi^ GC Tfh cells provide help to GC B cells and promote their survival, proliferation, and differentiation into immunoglobulin-producing cells (28). In human tonsils, GC Tfh cells contain subsets coexpressing Bcl-6 and RORγt, and Bcl-6 and T-bet (8). This suggests that other Th subsets may be able to differentiate into Tfh cells or that Tfh cells share the same developmental path with Th1 and Th17 cells as observed in mice (35, 36).

A CXCR5^+^ subset of CD4^+^ T cells can also be identified in the peripheral blood of mice and humans, subsequently referred to as circulating Tfh (cTfh) cells. Although these CCR7^lo^PD-1^+^ cells express lower levels of ICOS and PD-1 and seldom express Bcl6 they are closely related to Tfh cells (37). Their differentiation depends on ICOS and Bcl6 but not SLAM-associated protein (SAP), suggesting that cTfh cells are primary Tfh precursors or early memory Tfh cells. The cTfh cell subset undergoes active Tfh differentiation into mature Tfh cells in secondary lymphoid organs upon antigen reencounter and their presence correlates with autoimmune diseases such as lupus and rheumatoid arthritis (RA) (37). In addition, based on the expression of CXCR3 and CCR6, human cTfh cells can also be subdivided into three main subsets, namely, CXCR3^+^CCR6^−^ (Tfh1), CXCR3^−^CCR6^−^ (Tfh2), and CXCR3^−^CCR6^+^ (Tfh17) cells (38, 39). Tfh2 and Tfh17 cells, but not Tfh1 cells, represent efficient B cell helper cells to regulate immunoglobulin isotype switching (38). A recent data showed that in addition to cTfh memory, Tfh cells could be local in the draining lymphoid organs and sustain B cell responses after reactivation (40). In contrast to cTfh cells, local memory Tfh cells promote plasma cell differentiation and could be released to the circulating memory compartment over time.

## Tfh Cell Differentiation

T follicular helper cell differentiation is a complex process, which is tightly regulated (41) (Figure 1). It begins during naïve CD4^+^ T cell priming by DCs in the T cell zone of secondary lymphoid tissues and continues through the first cognate T cell:B cell interaction at the T–B junction until Tfh cells differentiate into mature GC Tfh cells when they enter follicles (2, 3, 42, 43). At each of these stages, Tfh cell development is influenced by signaling pathways downstream of cell surface molecules including the T cell receptor (TCR), costimulatory molecules, and cytokine receptors leading to the activation of specific transcriptional machinery (2, 3, 42, 43).

### Costimulatory Molecules

T follicular helper cell differentiation from naive CD4^+^ T cell precursors is a multistep process which requires costimulatory signals. Positive [CD28, ICOS, SAP, glucocorticoid-induced tumor necrosis factor receptor-related protein (GITR), etc.] and negative [CTLA4, PD1, and B and T lymphocyte attenuator (BTLA)] costimulation works together with TCR signaling during Tfh cell development to guide activation, proliferation, differentiation, migration, survival, and effector functions (1–3, 33, 44). Imbalance between positive and negative costimulation signals leads to increased Tfh cell number and consequently to Tfh-driven autoimmunity (45, 46).

#### Positive Costimulation

##### CD28 and ICOS

CD28 and ICOS are two structurally and functionally related costimulatory molecules that are critical for Tfh cell differentiation (2, 47, 48). CD28, which is constitutively expressed on both naïve and resting T cells, specifically binds to its ligands CD80 or CD86 and regulates T-dependent B cell responses (49, 50). While previous studies suggest the overlapping function of CD80 and CD86 in antibody responses, during virus infection CD86 expression on B cells but not CD80 is critical for Tfh cell generation and function (51). CD28 signaling regulates (i) early key events of Tfh differentiation, especially the expression of PD-1, ICOS, OX-40, Bcl6, and CXCR5 (47); (ii) the late stage of Tfh differentiation as B7 ligand blockade during ongoing infection impairs Tfh cell response (52, 53); and (iii) Tfh cell survival (53).

ICOS, another CD28 family member, is highly expressed on Tfh cells and is critical for Tfh cell generation and GC formation (2, 48). Unlike CD28, ICOS does not regulate any of the early differentiation steps of naïve T cells into Tfh cells, particularly Bcl6 expression (47); however, ICOS is important to maintain the phenotype of already differentiated Tfh cells (47). Mice in which the ICOS–ICOSL interaction is disrupted as well as ICOS-deficient patients have fewer Tfh cells and smaller GCs (6, 47). ICOS exerts its costimulatory function through phosphoinositide 3-kinase (PI3K) signaling which results in the activation of Akt (47). Akt phosphorylates the transcriptional factor FOXO1 which thereby stays in the cytoplasm and becomes functionally inactive (47). This is critical because FOXO1 suppresses Tfh cell differentiation through the negative regulation of Bcl6 and positive regulation of Klf2 expression (15). Klf2 plays a negative role in Tfh differentiation by binding to promoter regions of CXCR5, CCR7, CD62L, PSGL-1, and S1pr1, which leads to the suppression of CXCR5, relocation of Tfh cells from B cell follicles back to T cell zone and their phenotype reversion to non-Tfh cells (44). Besides the ICOS–Klf2 axis, ICOS promotes interaction of the p85a regulatory subunit of PI3K with osteoponin (OPN-i), followed by translocation of OPN-I into the nucleus, its interaction with Bcl6 and protection of Bcl6 from proteasome degradation; thus, sustain responses by Tfh cells (54).

##### SLAM-Associated Protein

SLAM-associated protein is an intracellular adaptor protein essential for the function of SLAM family receptors (SFRs) to regulate immune responses (55, 56). SFRs consist of nine members, four of which are highly expressed on Tfh and B cells: CD150/SLAM, CD229/Ly9/SLAMF3, CD84/SLAMF5, and NTB-A/Ly108/SLAMF6 (57–59). High intrinsic SAP expression in both human and mouse GC Tfh cells, but not in B cells, is important for the development of long-term humoral immunity: particularly formation of GCs, long-lived plasma cells, and memory B cells (57, 60, 61). Bcl6 is required for SAP expression in GC Tfh cells (62). SAP through SFRs regulates T:B cell adhesion, cytokine production, and TCR signaling strength (63). Patients with X-linked lymphoproliferative disease (XLP) caused by a SAP gene mutation as well as mice lacking SAP expression, display defects in GC Tfh cell generation and GC reactions (3, 55). While SAP-deficient CD4^+^ T cells express normal levels of CXCR5, ICOS, and Bcl6, they have an impaired ability to stably interact with cognate B cells and sustain GC reactions (58, 61, 64, 65). Thus, SAP is not required for early Tfh cell differentiation (Bcl6^+^CXCR5^+^), but is indispensable for sustained T:B cell interactions and the full polarization to GC Tfh cells (Bcl6^hi^PD1^hi^) mainly by the following mechanisms: (1) SAP binds phosphotyrosines of the SLAM immunotyrosine switch motifs (ITSM) and mediates positive signaling by recruiting Src family kinase Fyn and PKCθ (66); (2) SAP competes with the tyrosine phosphatase SHP-1 for Ly108 ITSM binding, signaling through which contributes to reductions of T:B contacts and to an impairment in GC Tfh cell generation and GC responses (58). In addition to regulating T:B adhesion, positive signaling through SAP and PKCθ regulates cytokine secretion by GC Tfh cells, particularly IL-4 (60, 67). Moreover, Sap/Ly108 engagement sustains the TCR signaling that is required for establishing the Tfh cell:B cell synapse and thus allowing Tfh cells to provide help to B cells (68).

##### Glucocorticoid-Induced Tumor Necrosis Factor Receptor-Related Protein

Glucocorticoid-induced tumor necrosis factor receptor-related protein (TNFRSF18, and CD357), a member of the tumor necrosis factor receptor superfamily, is expressed at high levels in Tregs and activated T cells (69). Activation of GITR by its natural ligand GITRL enhances proliferation and effector T cell responses and inhibits Treg mediated suppression (70, 71). GITR is highly expressed on Tfh cells compared with non-Tfh cells in the spleens of collagen-induced arthritis (CIA) mice (72). GITR signaling promotes Tfh cell expansion and survival. Administration of GITR-Fc protein greatly reduces CIA severity by suppressing Tfh development and thereby humoral immune responses. In addition, in the chronic lymphocytic choriomeningitis virus (LCMV) infection model, a Tfh cell intrinsic role for GITR in sustaining Tfh cell responses and LCMV-specific antibody production has been identified (73). Thus, GITR signaling is considered as a positive regulator of Tfh generation (44).

#### Negative Costimulation

##### Programmed Cell Death-1

Programmed cell death-1 (PDCD1 and CD279) is a member of the CD28 superfamily which is transiently expressed by activated conventional T cells and delivers an inhibitory signal by engaging its ligands PD-L1/CD274/B7-H1 and PD-L2/CD237/B7-DC that are expressed on activated B cells, DCs, and macrophages (74). PD1 shows high and sustained expression on exhausted T cells, Tregs, Tfh, and T follicular regulatory (Tfr) cells (75–78). In addition to conventional T cells, PD-1 is expressed by B cells, natural killer cells, and myeloid cells as well (79, 80). Early studies utilizing complete knockouts of PD1, PD-L1, or PD-L2, or their respective blocking antibodies tried to address the role of the PD-1 pathway in controlling humoral immunity. Interestingly, some studies have shown attenuated humoral immune responses upon PD-1 signaling blockade, whereas others have found enhanced responses (74, 81–85). Later work using cell-type specific deletion of PD1 and its ligand helped to delineate the function of PD1 signaling in GC responses and gain insight into the individual role of PD1 in Tfr and Tfh cells and PD1 ligands in DCs and B cells in the regulation of humoral immunity (77). The PD-1–PD-L1 pathway plays an immunoregulatory role in limiting the differentiation and suppressive function of Tfr cells (77). PD-1 expression can also modulate Tfh differentiation and function. Tfh cells from aged mice express higher levels of PD-1 compared with Tfh cells in young mice; PD-1 blockade in aged Tfh cells restores Tfh cell function, suggesting a cell-intrinsic role of PD1 in Tfh cells (12, 86). Interestingly, PD-L1 expression on DCs, but not B cells, inhibits Tfh and Tfr cell differentiation (87).

##### Cytotoxic T Lymphocyte Antigen 4

Cytotoxic T lymphocyte antigen 4 is a key checkpoint in immune tolerance (88–92). While CTLA4 and CD28 share the same ligands CD80 and CD86, CTLA4 interacts with them with higher affinity and avidity compared with CD28 (48). The central tenet of CTLA4 function is to regulate CD28 signaling, since fatal multiorgan inflammation as well as increased antibody levels in CTLA4 KO mice are prevented by the blockade of CD80 and CD86 (93–95). CTLA4 is constitutively expressed in Tregs and upregulated after activation in conventional T cells and plays a key role in mediating Treg function and in controlling conventional T cells (48). In fact, a Treg-specific deletion of CTLA4 recapitulates the phenotype of germline CTLA4 KO mice including increase in antibody production, indicating the role of CTLA4 in Tregs to control B cell responses (96). Recently, multiple groups assessed the role of CTLA4 in B cell responses and identified the function of CTLA4 on multiple T cell subsets including Tfh, Tfr, and Tregs in regulating humoral immune responses (13, 97). While it was suggested that CTLA4-dependent suppression is the primary mechanism used by Treg and Tfr cells to control Tfh cell development and humoral immunity *via* CTLA4-dependent downregulation of CD80 and CD86 on B cells, Foxp1-dependent CTLA4 expression on non-Treg CD4^+^ cells has cell-intrinsic and negative regulatory functions in Tfh cell differentiation, maintenance, and function (13). CTLA4 controls Tfh cell differentiation by regulating the degree of CD28 engagement (52).

##### B and T Lymphocyte Attenuator

B and T lymphocyte attenuator (CD272) is an inhibitory receptor expressed on T and B cells that binds TNFR family member herpesvirus entry mediator and attenuates T and B cell activation and effector functions (98–100). Mice lacking BTLA exhibit increased antigen-specific IgG responses and with age gradually develop autoimmune hepatitis-like disease and autoantibody production to nuclear antigens (101), suggesting that BTLA negatively regulates humoral immune responses. BTLA is highly expressed in CXCR5^+^ Tfh cells compared with conventional CXCR5^−^ CD4^+^ T cells. While Tfh cell development is not affected in BTLA-deficient mice, BTLA expression in Tfh cells but not in B cells is critical to control GC B cell development and antigen-specific IgG2a and IgG2b production (102). Moreover, BTLA controls Tfh-mediated B cell responses by suppressing IL-21 production (102).

### Cytokines

Along with antigen and costimulation signaling, specific cytokine-dependent cues play a central role in governing naive CD4^+^ T cell differentiation into specific effector T helper cell subsets. For example, IL-12 and IFNγ promote Th1 differentiation, whereas IL-4 drives Th2 differentiation (42). In addition, IL6 and IL-21 in combination with TGFβ induce Th17 differentiation (42). There are multiple cytokines that exercise either positive or negative roles at different stages of Tfh development (1, 2). However, cytokine-dependent Tfh cell formation varies between mice and humans (1, 42). Particularly, while TGFβ signaling opposes Tfh development in mice, it is required for human Tfh cell development (42).

#### Cytokines That Support Tfh Cell Formation in Mice and Humans

##### IL-6, IL-21, and IL-27

IL-6, IL-21, and IL-27 have all been implicated in Tfh cell development, although with differing roles (1, 2, 6, 7, 103, 104). IL-6 is mainly derived from activated B cells, DCs, and follicular DCs and is required in the initial stage of Tfh cell formation by inducing Bcl6 and IL-21 expression (5, 103, 105, 106). Mice deficient in IL-6 or IL-6R show reduced or delayed Tfh cell formation due to impaired signaling through STAT3 and STAT1 (5, 107). In addition, at the late stage of chronic viral infection, IL-6 derived from activated follicular DCs is crucial for maintenance of Tfh cell by upregulation of Bcl6 and viral control (3). Similar to mice, in humans, IL-6 derived from circulating plasmablasts is also a potent inducer of Tfh differentiation (108). IL-21 is primarily produced by select CD4^+^ T cells including Tfh, Th17 cells, and natural killer T (NKT) cells and plays a more prominent role in sustaining Tfh cell identity and function (6, 7, 18, 36, 109). IL-21- and IL-21R-deficient mice display reduced numbers of Tfh cells after antigen immunization suggesting an autocrine role for IL-21 in the maintenance and augmentation of Tfh cell programming (6, 110). However, in mice deficient either in IL-6 or IL-21 signaling, Tfh cell development is only partially compromised, indicating that these cytokines may play redundant roles in Tfh cell development (5, 103). In fact, loss of both cytokines significantly diminished Tfh cell numbers compared with an IL-6 or IL-21 deficiency alone (5, 103). However, an IL-6/IL-21 deficiency does not cause the complete absence of Tfh cells, suggesting an existence of IL-6 and IL-21-independent mechanisms for Tfh cell generation. In fact, it has been reported that the cytokine IL-27 contributes to Tfh cell maintenance by promoting IL-21 expression (104). Mice deficient in IL-27 signaling show reduced IL-21 expression, Tfh cell number, and GC activity (104). Similar to mice, DC-derived IL-27 is critical for the induction of Tfh cell polarization, IL-21 secretion by Tfh cells, and Tfh-dependent production of IgG by B cells (111). In addition to IL-21 induction, it has been suggested that IL-27 may play an important role in Tfh cell development by antagonizing IL-2 signaling, which negatively regulates Tfh cell development (10, 112).

##### TGF-β, IL-12, IL-23, and Activin A

Recent data suggest that different groups of cytokines support Tfh cell formation in humans, with prominent roles for TGF-β, IL-12, and IL-23. While IL-12 and IL-23 are capable of inducing IL-21 expression in naïve CD4^+^ T cells from human tonsils and peripheral blood, only IL-12 could augment expression of CXR5, ICOS, CD40L, and Bcl6, thus IL-12 is likely to act at an early stage of human Tfh cell development. B cells co-cultured with IL-12-primed CD4^+^ T cells produce antibodies which in part is dependent on the expression of CD40L (113). In support of the importance of IL-12 and IL-23 for Tfh development, individuals with mutations disrupting the function of IL-12Rβ1 (receptor for IL-12 and IL-23) have fewer circulating and GC Tfh cells and memory B cells. In mice, acting through STAT4, IL-12 induced a transitional stage in Tfh–Th1 cells, which express IL-21 and Bcl6. However, the IL-12–STAT4 pathway also promotes Tbet expression, which ultimately represses Bcl6 expression and Tfh cell programming. Since IL-12 is also linked to the generation of human Th1 cells, additional factors may also contribute to human Tfh cell generation. Surprisingly, TGF-β acts as an important cofactor for the early differentiation of human Tfh cells, but not in mice (7, 8, 114). TGF-β synergizes with IL-12 and IL-23, activating STAT4 and STAT3, and promoting Tfh cell development through induction of Tfh key molecules including CXCR5, ICOS, IL-21, Bcl6, Batf, c-Maf, and the downregulation of Blimp-1 expression (8). Recently, Activin A was identified as a novel inducer of Tfh cell programming in human and non-human primate cells, but not in mice by upregulating CXCR5, CXCL13, and PD-1 expression and repressing CCR7 and Blimp-1 expression (9). Activin A, in combination with IL-12, promotes the generation of Tfh like cells that have high expression levels of CXCR5, Bcl6, PD-1, LAMF1, CXCL13, IL-21, LIF, LTA, and TNF (9). Activin-A-induced Tfh programming is dependent on signaling *via* SMAD2 and SMAD3 (9).

#### Cytokines Which Inhibit Tfh Cell Formation

##### IL-2, IL-10, and IL-7

The IL-2/STAT5 axis is shown to be inhibitory for Tfh cell formation. In an *in vivo* mouse model of influenza infection, it was shown that exogenous administration of IL-2 suppresses Tfh cell differentiation, GC formation, and neutralizes antibody production (10). T cell-intrinsic expansion of Tfh cells is mediated by loss of IL-2Rα (115, 116). Mechanistically, activation of STAT5 by IL-2 enhances Blimp-1 expression and prevents binding of STAT3 to the Bcl6 locus (10). In addition, IL-2 inhibits the expression of IL-6 receptor α-chain (IL-6rα) and to a lesser extent, gp130 and thus negatively regulates Tfh differentiation (117). Moreover, a recent study depicts that IL-2 drives cells toward Th1 rather than Tfh differentiation through the activation of Akt and mTORC1 kinase (118). IL-7 negatively regulates Tfh differentiation by activating STAT5 and thereby repressing Bcl6 and CXCR5 (11). An additional study showed that IL-10, which has traditionally been viewed as a costimulator of antibody production, could also inhibit antibody responses indirectly by suppressing a subset of Tfh cells that produce IL-17 and IL-21 (119).

### Transcriptional Regulation of Tfh Cells

The differentiation of naïve CD4^+^ T cells into Tfh cells is regulated by coordinated interplay between cell-extrinsic factors and cell-intrinsic transcriptional networks. Since the discovery of Bcl6 as a key factor for Tfh differentiation, several other transcriptional factors that either support [IFN-regulatory factor 4 (IRF4), c-Maf, Batf, STATs, Ascl2, TCF1, LEF1, etc.] or oppose [FOXO1, FOXP1, BLIMP-1, STAT5, KLF2, and peroxisome proliferator-activated receptor gamma (PPARγ)] Tfh cell development, migration, and function have been defined (1–3). A dynamic balance between these multiple transcriptional factors is the main determinant of normal Tfh development and immunity, since dysfunction of negative transcriptional machinery triggers autoimmunity (120, 121).

#### TFs Positively Influencing Tfh Cell Differentiation

##### B-Cell Lymphoma 6 Protein

The Bcl6 has been recognized as a key transcriptional factor for Tfh cell development and for efficient GC responses (7, 26, 27). Expression of Bcl6 is driven by CD28 and IL-6/IL-21–STAT1/STAT3 signaling (47). Bcl6 overexpression leads to the upregulation of PD-1, CXCR5, CXCR4, and SAP which are essential for Tfh cell function in T and B cell interactions (106, 115). As a transcriptional repressor, Bcl6 functions to suppress genes that controls Tfh cell development by the following mechanisms: (i) Blimp-1 expression; (ii) genes encoding proteins that controls migration including EBI2, CCR7, CCR6, S1PR1, and PSGL1; (iii) Klf2 expression; (iv) genes that support Th1 (IFNGR1, T-bet, and STAT4), Th2 (Gata3), and Th17 (IL-17 and RORγt) cell development (7, 26, 27, 122–126).

##### c-Maf

c-Maf is a bZIP transcriptional factor that plays an important role in the regulation of cytokine production and Th2, Th17, and Tfh cell differentiation (87). Both Th17 and Tfh cells have higher expression of c-Maf, and loss of c-Maf in T cells results in a defect in IL-21 production and fewer Th17 and Tfh cells (36). Recent data further indicate an important and non-redundant role for c-Maf in the initiation of Tfh cell development and T-cell-mediated humoral responses. Loss of c-Maf expression in T cells leads to the decreased expression of key Tfh molecules, such as BCL6, CXCR5, and PD1 (127).

##### Batf

Batf is basic leucine zipper protein in the AP-1 family which was originally implicated in Th17 differentiation through direct regulation of transcription of RORγ, IL-21, and IL-22 (128). While Batf expression is moderately increased in Tfh cells compared with other T helper subsets, Batf is an essential component for Tfh development through combined regulation of Bcl6 and c-Maf (129, 130). Batf-deficient mice fail to generate Tfh cells, and Bcl6 and c-Maf overexpression in Batf-deficient T cells improves Tfh cell development but not to the level of Batf reconstitution, suggesting that additional targets are required for complete Tfh cell induction (130). In addition, the Batf/IRF4 complex in cooperation with STAT3 and STAT6 is required for IL-4 expression in Tfh cells (120).

##### IFN-Regulatory Factor 4

Together with the well-known function of IRF4 in Th2, Th17, Th9, and Treg differentiation as well in plasma cell maturation (131–139), IRF4 also plays a critical role in Tfh cell development and GC formation (140). Mechanistically, IRF4 may cooperate with other transcriptional factors that determine T cell fate decision: (i) IRF4 interacts with Jun and Batf to form an IRF4–Jun–Batf complex that binds to AP-1–IRF4 composite elements (141); (ii) binds with STATs (142), and Bcl6 (143). Recently, it has been acknowledged that TCR signaling strength controls IRF-4 concentration and consequently cell fate choice between Bcl6-expressing Tfh and Blimp-1 expressing Teff cells (144). Increased TCR signaling leads to elevated IRF4 levels that function to coordinate Teff cell fate choice at the expense of Tfh cell fate (144).

##### Signal Transducers and Activators of Transcriptions

There are several members of the STAT family including STAT1, STAT3, and STAT4 that contribute to Tfh cell development (1, 6, 14, 106). It has been reported that STAT1 is required for early Tfh differentiation (107). Besides, IL-6 signaling during Tfh differentiation is mediated by both STAT1 and STAT3 TFs (107). It has also been noted that STAT1 directly regulates expression of key Tfh genes including Bcl6, CXCR5, and PD-1 by binding to their promoter loci (145). IL-6–STAT3 and IL-21–STAT3 signaling can promote Tfh cell differentiation by inducing Bcl6 expression (6, 7, 146, 147). STAT3 cooperates with the Ikaros zinc finger TFs, Aiolos, and Ikaros, to regulate Bcl6 expression (148). IL-6-mediated STAT3 activation restricts IL-2Rα expression to limit Th1 cell differentiation (107). In humans, functional STAT3 deficiency compromises Tfh cell generation (149). STAT4 can promote the expression of Bcl6 and the classical Tfh cell cytokine IL-21 in both mouse and human Tfh cells *in vitro* (35, 150). However, continued IL-12-driven Stat4 signaling can decrease the expression of Bcl6 and IL-21 and strengthen T-bet-derived Th1 differentiation at the expense of Tfh cells (35). Interestingly after acute viral infection, T-bet is co-expressed with Bcl6 in Tfh cells and is required alongside STAT4 to coordinate IL-21 and IFN-γ production in Tfh cells and for promotion of the GC response (151).

##### Notch1 and Notch2

Notch proteins belong to the family of evolutionary conserved transmembrane-bound receptors and play an important role in CD4^+^ T helper cell differentiation and/or function including Tfh cells (152). Mice with T-cell-specific deletions of Notch1 and Notch2 display impaired Tfh cell differentiation, IL-4 secretion by Tfh cells and GC reactions after immunization with T-dependent antigens or infection with parasites (153). Notch1- and Notch2-deficient Tfh cells express reduced levels of Tfh-associated molecules (CXCR5, PD-1, BTLA, and Bcl6), but normal levels of ICOS and increased Blimp-1 expression (153). Notch receptors 1 and 2 are required for Tfh cell generation and IL-4 expression by Tfh cells, but are dispensable for Th2 cell differentiation in response to parasitic helminth infection. Thus, Notch signaling is an important checkpoint in the bifurcation between Tfh and Th2 cell-driven hallmarks of type-2 immunity (154).

##### NFAT

Nuclear factor of activated T cells 2 (NFAT2) is highly expressed in Tfh cells, NFAT2 deficiency in T cells leads to enhanced GC reactions due to the impairment of Tfr cells to upregulate CXCR5 but not Tfh cells (155). A loss of both NFAT1 and NFAT2 in CD4^+^ T cells leads to impaired GC reactions due to reduced Tfh cell differentiation and decreased expression of proteins such as ICOS, PD-1, and SFRs which are important players in T/B interactions and B cell help (156).

##### Achaete-Scute Homolog 2

Achaete-scute homolog 2, a bHLH-domain-containing TF, is selectively upregulated in Tfh cells and initiates Tfh cell development (157). Overexpression of Ascl2 can lead to a substantial induction of CXCR5 expression, but not Bcl6 and downregulation CCR7 expression *in vitro*, as well as accelerated T cell migration to the follicles and Tfh cell development *in vivo* in mice (157). Ascl2 inhibits expression of Th1 and Th17 signature genes (157). Ascl2 deletion as well as inhibition of its function with E-protein inhibitor Id3 leads to a total impairment of Tfh cell development and GC response (157).

##### T Cell Factor 1 (TCF-1) and LEF-1

T cell factor 1 is highly expressed in Tfh cells (158–160). TCF-1 plays an important role in the initiation of Tfh cell differentiation and the effector function of differentiated Tfh cells, as TCF-1 deficiency results in reduced generation of Tfh cells and impairs their function to provide B cell help (159, 160). Similar to TCF-1, LEF-1 is also known for its essential role in early Tfh cell development (161, 162). LEF-1 and TCF-1 are upstream of Bcl6 induction and directly target Tfh signaling molecules (Bcl6, IL-6R, gp130, and ICOS) to promote Tfh cell differentiation (158). TCF-1 also suppresses Blimp-1 and IL-2Ra expression (159).

##### Early Growth Response Gene 2 (EGR2) and EGR3

Early growth response gene 2 and EGR3 can directly regulate the expression of Bcl6 and differentiation of Tfh cells (163).

##### Bob1

It has been reported that B-cell-specific octamer-binding protein 1, Bob1 in cooperation with TFs Oct1/Oct2 can directly bind to Bcl6 and BTLA promoters and promote their expression and Tfh cell development (164). However, at the same time, other groups have reported that the function of Bob1 is to mainly restrict the cellular frequency of Tfh cells (165, 166).

##### NF-kB1

It has been reported that NF-kB1 promotes Tfh cell responses by facilitating CXCR5 expression but no other Tfh-related molecules (Bcl6, IL-21, and PD-1), and the NF-kB1-deficient T cells partially lose their ability to provide help to B cells *in vivo* (167). In addition, the non-canonical NF-kB pathway may also play an essential role in Tfh development through regulation of ICOSL expression in B cells (168).

#### TFs Negatively Influencing Tfh Cell Differentiation

##### FOXO1 and FOXP1

Early studies suggest that a decreased expression of FOXO1 either because of increased expression of ICOS (15) or because of ITCH-mediated degradation (169) may increase Tfh cell differentiation. Analysis of mice with a specific deletion of Foxo1 in T cells revealed the requirement for Foxo1 in the suppression of Bcl6 expression and Tfh cell differentiation (15). In addition, enforced nuclear localization of Foxo1 prevents Tfh cell differentiation (15). However, Foxo1 is required during final differentiation to GC Tfh cells as Foxo1 deficient GC Tfh cells are substantially reduced (15). Foxp1 is an additional negative regulator of Tfh cell development. It mainly plays a role in dampening ICOS and IL-21 expression by regulating CCR7 and CTLA4 expression (170).

##### Krüppel-Like Factor 2

The TF, Klf2, regulates naïve T cell trafficking to secondary lymphoid tissues by promoting the expression of CD62L and S1PR1 (16). KLF2 expression impairs Tfh cell differentiation, whereas ablation of KLF2 expression enhances Tfh cell differentiation (171). This effect is related to the capacity of KLF2 to promote the expression of genes that oppose Tfh cell differentiation (Blimp-1, Gata3, and T-bet) and to repress the transcription of CXCR5 (171).

##### Blimp1 and STAT5

Blimp1, which is encoded by *Prdm1*, is a transcriptional repressor that has the ability to inhibit Bcl6 expression in B and T cells (122). Blimp-1, induced by IL-2/IL-7 and STAT5 signaling, suppresses expression of Bcl6 and other Tfh-associated genes including CXCR5, c-Maf, Bcl6, Batf, and IL-21, thus preventing Tfh cell differentiation (14, 172).

##### Peroxisome Proliferator-Activated Receptor Gamma

Peroxisome proliferator-activated receptor gamma is a TF that regulates lipid and glucose metabolism (173). One-year-old T-cell-specific PPARγ-deficient mice exhibited a moderate autoimmune phenotype with increased Tfh cells, GC B cells, glomerular inflammation, and enhanced autoantibody production. Mechanistically, PPARγ by stabilizing the activity of Ikbα, Foxo1, and Sirt1 negatively regulates Bcl6 and IL-21 to inhibit Tfh cell differentiation and GC formation (173).

## Other Follicular T Cells

Several types of follicular T cells that have been found in GCs and characterized such as Tfr cells, follicular regulatory CD8^+^ T cells (CD8 Tfr), natural killer T follicular helper (NKTfh) cells, and follicular CD8^+^ T cells (fCD8).

### Tfr Cells

T follicular regulatory cells, a newly identified subset of Tregs, have been found in GCs where they control GC responses (75–77, 86, 174–176). Tfr cells originate from thymic-derived Foxp3^+^ T cells as well as from Foxp3^−^ precursors rather than from Tfh cells (78, 177). They are different from Tfh cells and Tregs: on the one hand, Tfr cells express large amounts of Tfh-related factors including CXCR5, PD-1, Bcl6, CXCL13, and ICOS; on the other hand, they share numerous molecules that are expressed by Tregs, such as GITR, CTLA4, IL10, CD25, and Foxp3 (75, 76, 178).

T follicular regulatory cells similarly possess a multistage and multifactorial differentiation process (176) that requires CD28 and ICOS signaling (76, 77), Sap-dependent interaction with B cells (76), and expression of Bcl6 (178). TF NFAT2 contributes to the initial upregulation of CXCR5 in Tfr cells (155). In addition, recently it has been noted that the mTORC1–STAT3–TCF-1–Bcl6 axis and TRAF3 are essential for Tfr differentiation (179, 180). However, there are also some signals which inhibit Tfr-cell differentiation and function: PD-1, CTLA4, Blimp1, and helix-loop-helix proteins ID2 and ID3 (77, 78). In addition, the cytokine IL-21 can suppress Tfr cells through the upregulation of Bcl6 expression and downregulation of CD25 (103, 181, 182).

T follicular regulatory cells act to limit excessive GC responses by acting on both Tfh and GC B cells (13, 77, 86, 183). Moreover, Treg suppression is not limited to GC B cells and occurs at various steps during B cell differentiation, from B cell activation to class-switched B cells and plasma cells (78). The effect of Tfr cells to modulate GC reaction could be through several potential mechanisms. Tfr cells could suppress Tfh cells through CTLA-4, by dampening expression of CD28 ligands on GC B cells (13). Two immunosuppressive cytokines, IL-10 and TGF-β, may mediate the immune suppressive functions of Tfr within GCs (114, 119, 176, 184); however, a recent research showed that IL-10 is important for B cell survival and proliferation; thus, a detailed mechanism remains to be investigated (185). Interestingly, Tfr cells potentially have effects on antibody affinity, and their suppressive function could result in the selection of the highest affinity antigen-specific antibody and in the selection of higher affinity memory B cells (78).

### Follicular Regulatory CD8 T Cells (CD8 Tfr)

In contrast to Foxp3^+^CD4^+^ Tregs, mouse CD8 Tregs do not constitutively express Foxp3 in the thymus and periphery (186), and the CD8^+^Foxp3^+^ T cells do not comprise CD8 Treg population due to lack of suppressive activity (187). Similarly, most of the human CD8 Tregs also lack Foxp3 (188). In mice, a specific subset of Qa-1-restricted CD8 Tregs with high expression levels of CXCR5 (named as CD8 Tfr) were found to possess the ability to limit GC size and prevent autoimmune disease in mice (189). Tfh cells are one of main targets of CD8 Tfr cells (189). In autoimmune-prone mice, CD8 Tfr cells can suppress the expansion of Tfh cells as well as autoantibody production (190). Recent data indicate on the importance of the TF STAT4 for CD8^+^ Tfr development, maintenance, and function toward Tfh and plasma B cells (191). Moreover, CD8 Tfr cells expressing IL-2Rβ are also shown to inhibit CD8 T cell function in an IL-10-dependent manner (192). Recently, cells with a CD8 Tfr phenotype (CD3^+^CD8^+^CXCR5^hi^CD44^hi^) have been identified in humans (193). In chronic CIV infection, CD8 Tfr cells localized in the follicles exhibit enhanced Tim-3 and IL-10 expression, but express less perforin compared with CD8 T cells. CD8 Tfr cells modestly limit HIV replication in Tfh cells by impairing IL-21 production *via* Tim-3 and inhibit B cell function (194). In addition, it has been reported that the KIR^+^CD8^+^ cells (KIR, killer cell immunoglobulin-like receptor, functional homolog of murine Ly49) exert inhibitory activity on CD4^+^CXCR5^+^ Tfh target cells in humans (193).

### NKTfh Cells

Recently, a subset of invariant NKT cells, recognized as follicular helper NKT cells (NKTfh cells), was discovered (195). NKTfh cells express CXCR5, PD-1, and Bcl-6 and support B cell responses (196). Similar to the development of conventional Tfh cells, the formation of NKTfh cells is dependent on CD28-mediated cognate interactions with B cells and Bcl6 expression (196). Studies utilizing CD4^+^ T cell-specific loss of Bcl6 determined that both Tfh and iNKTfh cells contribute to B cell help (197). However, unlike Tfh-derived B cell responses, those driven by NKTfh cells have no potential to generate long-lived plasma cells and memory B cells (196). NKTfh cells also possess the ability to boost memory B cell responses to T-dependent antigens but not T-independent lipid antigens (198). In addition, the NKTfh cells can induce limited GC B cell responses in the absence of CD4^+^ cell help (199). Further studies are still needed for a deeper understanding of the mechanisms of NKTfh cells in immunity and their roles in disease.

### Follicular CD8^+^ T Cells (fCD8)

In contrast to the previous opinion that CD8^+^ T cells are restricted to extrafollicular areas (200), recent studies have showed that the CD8^+^ T cells from human and non-human primates possess the ability to migrate to the lymphoid follicles and GCs to support B cells (201). CD8^+^ T cells localize in human tonsil follicles and possess follicular helper-like characteristics including high expression of Bcl6, CXCR5, ICOS, PD-1, CCR5, CD27, CD28, CD69, and CD95, but do not express CCR7, Blimp-1, Tim-3, and CD244 and are named as follicular CD8^+^ T cells (fCD8) (201–206). The current evidence suggests a crucial role of fCD8 in controlling intracellular pathogens and malignancies through the production of various cytokines (IFN-γ, TNF-α, and MIP1β) in LCMV-infected mice, HIV-infected individuals, and cancer patients (203–205, 207–211). These cytokines activate APCs, promote polarization of naïve CD4^+^ T cells to Th1 cells, sustain activation of CD8^+^ T cells; thus, contributing in the control of viral infection and tumor growth. In addition, similar to Tfh cells, IL-21-producing fCD8 cells promote Ag-specific antibody responses by stimulating B cells, as well as generating and maintaining follicles and GCs (201).

## The Role of Tfh Cells in Diseases

**Figure 2:**
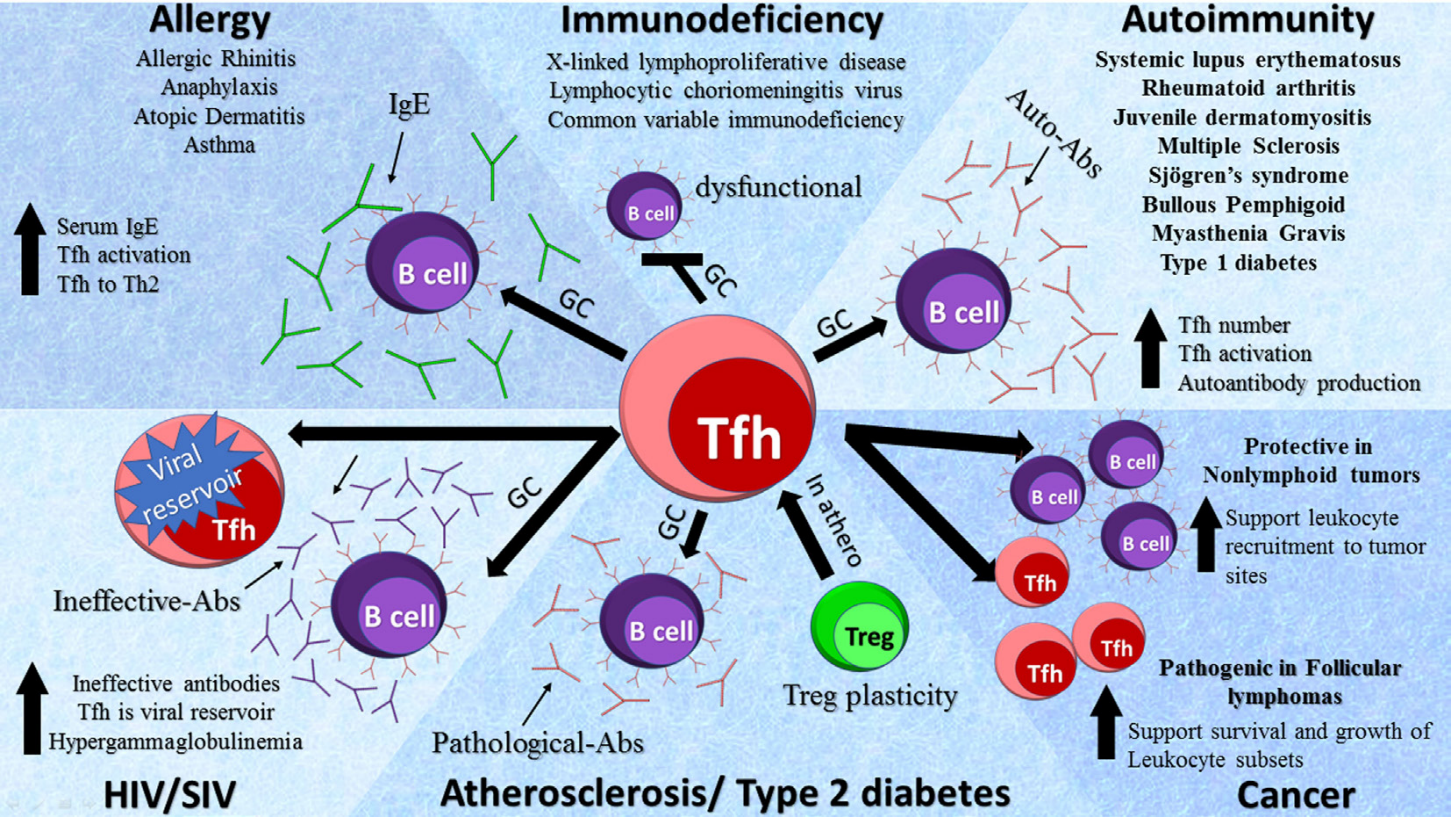
Role of T follicular helper (Tfh) cells in diseases. Tfh cells are involved in a variety of pathologies by diverse mechanisms as depicted in the schematic diagram. Elevated Tfh activity leads to an increase in germinal center (GC) formation, activity, and subsequent production of auto-Abs in autoimmune diseases. In primary immunodeficiencies, a decrease in Tfh cells has been detected with dysfunctional GC formation, whereas in acquired immunodeficiencies such as HIV and SIV an increase in Tfh cells serves as viral reservoirs. In atherosclerosis regulatory T cells (Tregs) transition to Tfh cells *via* GCs leading to the production of pathological Abs. In allergy, there is an increase in Tfh cell activation and GC formation causing the production of IgE. The role of Tfh cells in cancers is complex and dependent on the types of tumors.

The main function of Tfh cells is to control clonal selection of GC B cells and support B cell immunoglobulin synthesis, isotype switching, and somatic hypermutations. Pathological B cell activation and the production of autoantibodies is a hallmark of the defective immune response that accompanies autoimmunity. In the sections below, we discuss the role of Tfh cells in various disease settings (Figure 2).

### Systemic Lupus Erythematosus (SLE)

Observations in animal models and in humans provide strong evidence that Tfh cells are important players in SLE pathology. Patients with SLE have an increased number of cTfh cells, which positively correlates with autoantibody titers (212). Furthermore, the proportion of peripheral blood T cells expressing ICOS is higher in patients with active SLE disease than in patients with inactive disease or in controls which probably leads to enhanced autoantibody production upon activation *via* ICOS–ICOSL interactions in these patients (45, 213). Production of high levels of IL-21 is a hallmark of Tfh cells and multiple studies have shown an increased frequency of CD4^+^IL-21^+^ T cells in SLE patients that was associated with disease severity (214). An increase in Tfh cells is also associated with a shift in the Th17/Treg populations with increases in Th17 cells and decreases in Tregs as well as an elevation of IgG^+^ class-switched memory B cells leading to a more inflammatory environment as observed in SLE (214–219). It is worthy to note that Tfh cells not only affect priming in secondary lymphoid tissues but can also impact local B cell activation and expansion. For example, within the tubulointerstitium of patients with lupus nephritis, Tfh-like cells are organized with B cells in structures resembling GCs (220, 221) suggesting that Tfh cells may also play a role in various complications that are associated with autoimmune pathologies.

Mice that have a single-amino-acid mutation in Roquin1 (a negative regulator of ICOS mRNA stability) demonstrate a spontaneous lupus-like phenotype that is accompanied by elevated numbers of Tfh cells expressing higher level of ICOS, IFN-γ OX40, and IL-21 and activated phenotypes of GCs (28, 222). Importantly, manipulation of Bcl6 expression and thus Tfh cell generation or adoptive transfer of lupus-associated Tfh cells from Roquin^san/san^ mice into healthy recipients induces the formation of GCs, highlighting a contributing role of Tfh cells in SLE (223). Another interesting model to study lupus-related pathophysiology is the BXSB mouse model, which displays lymphoid hyperplasia, monocytosis, immune complex-mediated glomerulonephritis, and an aberrant IL-21-dependent Tfh response (224). The MRL/lpr mouse model displays defective Fas signaling (225), which is characterized by high levels of autoantibodies and extrafollicular Tfh like cells that are dependent on ICOS, Bcl-6, and IL-21 signaling. The pathological functions of these T cells support extrafollicular B cell differentiation and plasmablast maturation (226). While increasing evidence suggests the importance of an extrafollicular Tfh-dependent response in the murine models of SLE (227–230), the importance of Tfh cells and extrafollicular sites in SLE patients is not well defined due to limitations in obtaining non-circulated follicular-resident Tfh cells.

There have been several relatively successful attempts to reduce the severity of SLE in humans *via* blockade of Tfh-cell differentiation and activity. Early results from SLE therapies targeting T cells showed that patients had reduced serum anti-dsDNA titers, but presented minimal reduction of protective antibodies, increased complement markers, and reduced nephritis scores (231). Studies using monoclonal antibodies against ICOS-L inhibited the development of Tfh and GC B cells resulting in decreases in anti-dsDNA Igs and improved kidney function. For years, the main therapy for SLE has been broad spectrum immunosuppressant’s; however, a growing body of work shows that Tfh cells may be a more precise and attractive target for treating SLE.

### Sjögren’s Syndrome

Increased numbers of human peripheral blood CXCR5^+^ICOS^+^, CXCR5^+^PD-1^+^ Tfh cells and enhanced GC formation positively correlate with autoantibodies titers and severity of the primary Sjögren’s syndrome (pSS) (227–230). IL-6 levels are increased in the serum, tears, and salivary gland epithelial cells of patients with pSS. Interestingly, co-cultures of salivary gland epithelial cells with T cells induce Tfh cell differentiation (3, 232) suggesting a critical role for epithelial cell-derived IL-6 in Tfh cell differentiation in pSS. There are several treatment options for patients with pSS that target Tfh cell functions and therefore the humoral response. Abatacept is a fusion molecule combining CTLA-4 with IgG Fc that binds to CD80/86 and consequently impairs CD28-mediated T cell costimulation (233). It has been shown that abatacept inhibits T cell-dependent B cell activation either *via* DC-defective Th activation or *via* blockade of T/B cell interactions (234). Abatacept treatment in pSS patients reduces circulating numbers of ICOS^+^ cTfh cells resulting in an attenuated Tfh cell-dependent B cell hyperactivity (235) providing a promising therapy for pSS and some other autoimmune pathologies.

### Juvenile Dermatomyositis (JDM) and Autoimmune Myasthenia Gravis (MG)

Serum autoantibodies can be found in up to 70% of patients with JDM. Furthermore, these patients display changes in cTfh cells subsets with a decrease in cTfh1 cells, and an increase in the cTfh2 and activated memory cTfh17 cell subpopulations; resulting in an overall increase in cTfh subsets with efficient helper functions. Significantly, these changes in the composition of cTfh cell subsets positively correlate with disease activity and the frequency of circulating plasmablasts (38).

Elevated levels of circulating CXCR5^+^CD57^+^, CXCR5^+^ICOS^hi^, and CXCR5^+^PD1^hi^ CD4^+^ T cells have also been reported in patients with MG (236). Functional studies demonstrate that cTfh cells from MG patients support autoantibody production, and thus, contribute to the development of disease. Interestingly, Tfh1 and Tfh17, but not Tfh2 cells, were found to be the major secretors of IL-21 (237). Thus, alterations in the composition of peripheral blood memory Tfh1, Tfh17, and Tfh2 subsets seems to be one of the distinct features of several autoimmune diseases including SLE (238), Sjogren’s syndrome, multiple sclerosis (239), and MG (237). While an increasing body of evidence reports a shift in the Tfh1/Tfh2/Tfh17 balance in the peripheral blood of patients with autoantibody-mediated pathologies, it is not clear whether the same shift in the Tfh subsets occurs within the inflamed tissues and GCs in the secondary lymphoid tissues. It would be important to identify molecular mechanisms and microenvironmental stimuli that direct a preferential repopulation of Tfh17 and Tfh2 cells in autoimmune settings.

### Rheumatoid Arthritis

One of the other examples for the implication of Tfh cells in autoimmune disease setting is RA, which is characterized by high levels of autoantibodies and abnormal GC B cell responses, which contribute to inflammation in the joints. Tfh cells have been detected in the synovial tissue of patients with RA, and along with IL-21 are found in higher frequencies in the periphery (240–242). The increase in Tfh cells in RA patients is positively correlated with elevated serum level of anti-CCP antibodies and consequently with disease severity (240). In animal models, a T cell-specific CXCR5 deficiency results in a significant reduction in GC formation, decreased levels of collagen-specific IgG1 antibodies and a resistance to CIA induction (243). As Tfh cells play a significant role in the progression of RA, therapeutic targeting of Tfh cells could be a valuable option for treating patients with RA.

### Type 1 Diabetes (T1D)

Type 1 diabetes is caused by the autoimmune destruction of insulin-producing β-cells in the pancreas. T1D patients have higher frequencies of circulating CD4^+^CXCR5^+^ICOS^+^Tfh cells and higher levels of IL-21 which positively correlates with an increase in plasmablasts, serum autoantibodies, and C-peptide levels (244–246). It is possible that Tfh cells may play a critical role in the initial stages of T1D with an increase of activated CXCR5^+^PD-1^+^ICOS^+^ cTfh cells being found in both children with newly diagnosed T1D and in children at late stages of preclinical T1D, characterized by impaired glucose tolerance (247, 248). Data from animal models also show that transfer of Tfh cells from a diabetic to a control animal induces elevated blood glucose levels and increased T cell infiltration into islets (244). Furthermore, Roquin^san/san^ mice that have overreactive Tfh cells display dramatically accelerated T1D induction. Collectively, there is direct evidence for a pathogenic role for Tfh cells in progression to T1D. Importantly, the increase in activated cTfh cells is strongly associated with positivity for multiple autoantibodies and could be used as a biomarker for the identification of a subgroup of patients with an active setting of T1D.

### Type 2 Diabetes (T2D)

Evidence indicates a key contribution of the immune response in the manifestation of chronic low grade inflammation under the conditions of adipose tissue inflammation, islet β-cell dysfunction, and T2D. While the role of macrophages, CD4^+^, CD8^+^ cells, and follicular B cells is firmly established, there is limited knowledge about the Tfh cell role in obesity and T2D. Recently, Zhou and colleagues found that non-obese T2D patients vs BMI-matched healthy subjects have higher IgG levels and Tfh cells that are highly enriched in IFN-γ, but not IL-4 and IL-17 (249). Interestingly, overweight T2D patients (BMI ≥ 24.0) had higher levels of cTfh and the balance of cTfh cell subsets was shifter toward the Th17 subtype (52). Importantly, patients with abdominal obesity had additional increases in cTfh compared with patients without abdominal obesity (52), suggesting that cTfh may play a critical role in the modulation of adipose tissue inflammation in obesity-induced T2D.

### Atherosclerosis

Recent reports revealed a critical role for Tfh cells and consequently GC B cells in the development and progression of human and mouse atherosclerosis. Tfh cells support atherogenesis *via* the production of pathological Abs and the generation of highly active GCs. The loss of Tfh cells *via* a conditional knockout of *Bcl6* leads to a reduced atherosclerotic burden in atherosclerosis-prone mice (250, 251). Interestingly, a percentage of Tregs can switch the phenotype into pro-atherogenic Tfh cells but ApoAI can prevent Treg to Tfh cell conversion throughout atherosclerosis (250). It remains to be determined how atherosclerosis-prone conditions alter the memory pool of Tfh cells and how Tfh cells impact the processes of selection of high-affinity B cells and B cell memory development in atherosclerosis.

### Allergy

Patients with allergic rhinitis (AR) and asthma preferentially have increased levels of cTfh cells that exhibit a Tfh2 cell phenotype (252). In patients with seasonal AR to ragweed pollen, activation of Tfh cells increases significantly during peak-season (253). It has been noted that the acute anaphylaxis response to peanut allergen is driven by IL-4^+^IL21^+^ Tfh cells and to a lesser extent by Th2 cells (254). Presence of Tfh cells but not Th2 cells is required for IgE production and for the development of an allergic response.

The increased levels of serum IgE is also a hallmark of atopic dermatitis (AD) (255–258). Peripheral Tfh cells in children with AD have significantly increased levels of ICOS, PD-1, and IL-21 suggesting a highly activated phenotype. Furthermore, a strong positive correlation has been detected between the numbers of IL-21^+^ Tfh-like cells, activated memory B cell pool, and disease severity (259, 260). Interestingly, patients with asthma also display elevated levels of PD-1^+^ICOS^+^ Tfh2 cells and the ratio of Tfh2:Tfh1 cells positively correlates with the total IgE levels in the blood (46). In correlation with the data on human samples, mRNA and protein expression levels of CXCR5, ICOS, ICOSL, and IL-21 were also elevated in mouse models of asthma. Our recent findings also identify an important role of the TF Batf toward the generation of IL-4-expressing Tfh cells rather that Th2 and to their pro-allergic function (120). We further demonstrated that the IL-4–STAT6 signaling contributes to the Batf induction in Tfh cells and the Batf/IRF4 complex along with Stat3 and Stat6 aids IL-4 production in Tfh cells. Recent studies have also shown that Tfh cells can sense specific microenvironmental conditions and differentiate into Th2 cells after repeat exposure with house dust mite (HDM) (261). At the initial stage of disease, Tfh cells are preferentially differentiated, but a second exposure leads to the Tfh switch into Th2 cells which migrate to the lung and produce an inflammatory response. These results suggest that targeting Tfh cells may be a good therapeutic strategy to prevent Th2-cell-mediated immunity to HDM (261).

### Primary Immunodeficiency

There are several immunodeficiencies that directly affect the development and functions of Tfh cells and as a result alter B cell-dependent responses. XLP is a primary immunodeficiency caused by mutations in SH2D1A (encoding for SAP, signaling lymphocytic activation molecule-associated protein). A SAP deficiency does not impact CD4^+^ T cell development, but compromises Tfh cell differentiation (262). In line with this observation, XLP patients exhibited significant defects in GC formation, reductions in memory B cell responses, hypogammaglobulinemia, and impaired antigen-specific antibody responses (263). Mutations in CD40L, ICOS, and STAT3 also cause reduced number of CD4^+^CXCR5^+^ cells, defective GC formation, and impaired humoral immune responses. A recent study also highlighted the importance of the IL-12/IL-12R axis as patients with mutations in IL-12Rβ1 demonstrate fewer circulating memory Tfh, memory B cells, and defective GCs compared with control subject (264).

### HIV and SIV Pathologies

Proper activation of Tfh cells and their interactions with GC B cells are essential for an effective humoral immune response and the extermination of pathogenic human and simian immunodeficiency viruses (HIV and SIV, respectively). Paradoxically, HIV-infected individuals (265) and monkeys infected with SIV (266) display a significantly higher frequency of ICOS^+^ Tfh, PD-1^+^ Tfh, and ICOS^+^PD-1^+^ Tfh cells among total CD4^+^ T cells compared with non-infected controls. Furthermore, Tfh cell frequency is significantly higher in non-treated HIV^+^ patients compared with HIV^+^ patients treated with combination anti-retroviral therapy, suggesting that an HIV viral persistence contributes to Tfh cell expansion (265–267). Interestingly, Tfh cells in humans and macaques show preferential infection with HIV and SIV, respectively (200, 267, 268), likely *via* the chemokine receptor CCR5 as CCR5 is expressed on a precursor subset of Tfh cells and may potentially serve as a co-receptor for HIV (269). GC Tfh cells have been implicated in HIV persistence by supporting viral replication during treated infection and serve as an important cellular reservoir of HIV-1 DNA (270, 271). One explanation is that cytotoxic CD8^+^ T cells are CXCR5 negative and thus unable to migrate to follicles and target HIV-infected GC Tfh cells (200, 272, 273). In line with an increased number of Tfh cells, hypergammaglobulinemia is detected in HIV^+^ patients (274), but these antibodies are ineffective (265, 273, 275–278). Studies have proposed that PD-1 triggering by PD-L1 on GC B cells is a mechanism for the abnormal Tfh functions and defective B cell help (277). In addition, due to the increased number of Tfh cells, the exacerbated interactions of Tfh and GC B cells may lower the threshold for B cell selection resulting in the selection of B cells with low affinities (223). Thus, the relationship between viral entrance into Tfh cells, number and functions of Tfh cells, and B cell activation/maturation is complex and requires further investigations.

### Chronic Infections

Lymphocytic choriomeningitis virus persistence results in extended TCR engagement and an IL-6-driven shift from a Th1-induced response toward a Tfh response (279, 280). In addition to studies that identified a key role of Tfh cells in HIV and SIV infections, studies with the LCMV disease model further highlight the importance of timely expansion and sustained functions of Tfh cells in the orchestration of a proper humoral response for control of persistent viral infection (279, 280). Moreover, annual influenza virus studies determined an increase in the number and activation levels of cTfh cells. These studies not only show that an increased cTfh cell frequency contributes to circulating plasmablast responses and infection clearance but can also be used as a marker to monitor the efficacy of influenza vaccination (281, 282). In other infections such as chronic hepatitis B virus patients have an increase in circulating regulatory Tfh cells (283). Interestingly, long-term disruptions of proper T cell-dependent Ab production have been detected in the case of parasitic infections such as *Leishmania* (284), *Litomosoides sigmodontis* (285), and *Plasmodium* infection (286) suggesting the negative regulation of the number and functions of Tfh cells under these conditions. To date, the precise mechanisms that regulate differentiations and functions of Tfh and Tfr cells in different infection settings remain to be determined. Particularly, further studies are required to assess the mechanisms governing Tfh cell development, persistence, and function at the different stages of various infection diseases.

### Cancer

Accumulating evidence suggests that Tfh cells are involved in peripheral T cell and B cell-associated tumors due to their high impact on growth and survival of different leukocyte subsets. Angioimmunoblastic T cell lymphoma (AITL) is an aggressive tumor and isolated neoplastic T cells express CXCL13, ICOS, CD154, CD40L, and NFATC1 (287, 288), making these T cells similar to Tfh cells (289). The expression of mutated RhoA G17V induces Tfh cell differentiation/activation; increased proliferation associated with ICOS upregulation, elevated PI3K, and mitogen-activated protein signaling (290). Loss-of-function mutations in epigenetic regulators such as TET2 and DNMT3A are frequent events in the pathogenesis of AITL. Interestingly, RhoA G17V expression accompanied with TET2 loss results in AITL development in mice. It is worth to note that altered RhoA GTPase activity has been linked with autoimmunity and studies in AITL further highlight a role of RhoA in shaping Tfh cell phenotype and response.

Like AITL, in follicular T cell lymphomas, infiltrating T cells resemble a phenotype of Tfh-like cells and express IL-4, TNF-α, IFN-γ, LT-α, CCL17, and CCL22 chemokines that play a role in the regulation of Treg and Th2 cell migration and modulate the activity of GC B cells within follicles as well. Not only Tfh-like cells but also Foxp3^+^ Tfr cells are found within neoplastic follicles and the number of Tfr cells is elevated during progression lymphomagenesis. Thus, there is a complex T cell response that is regulated by a delicate balance of CD4^+^, Tregs, and Tfh subsets. Probably one of the strongest predictors of survival would be the location pattern for T cell subsets, as accumulation within the follicles is linked with poor survival compared with a distribution pattern outside the follicle (291). To date, there is limited understanding in the functions of Tfh and Tfr subsets in lymphomagenesis and more detailed research in animal models and human samples will help to dissect the complex role of Tfh cells in cancer.

Unexpectedly, Tfh cells have protective roles in nonlymphoid tumors. Higher levels of Tfh cell infiltrates and their ability to organize tertiary lymphoid structures within tumors has been associated with increased survival and reduced immunosuppression which strongly correlate with an increased survival in breast cancer (292). Evidence suggest that IL-21 and CXCL13 may play a key role in the protective functions of Tfh cells *via* the modulation of local leukocyte recruitment. Infiltrating Tfh cells have also been reported in chronic lymphocytic leukemia, non-small cell lung cancer, osteosarcoma, and colorectal cancer (214, 293–297), where they positively correlated with patient survival (293). So far, very little is known about how Tfh cells impact the immune response involved in the suppression of tumor initiation and progression and further studies will be important for a better understanding of Tfh-related pathologies.

## Conclusion

Since the identification of Tfh cells and the discovery of Bcl6 as a critical factor in their generation, there has been substantial progress made in understanding the molecular and cellular requirement for the development and function of mouse and human Tfh cells. To date, multiple cell-extrinsic and -intrinsic factors (costimulatory molecules, cytokines signaling, and transcriptional factors) have been determined to positively or negatively contribute to Tfh cell development. However, still many questions remain to be answered: (i) What is the exact composition and hierarchy of these factors and what are their stage-specific requirements? (ii) Is Bcl6 required complete Tfh cell commitment? (iii) How Tfh-specific transcriptional factors impact epigenetic mechanisms governing Tfh cell generation? (iv) Which factors contribute to Tfh cell maintenance and memory formation? (v) What are the appropriate Tfh-specific target(s) for therapy in Tfh mediated autoimmune disorders and cancer?

T follicular helper cells mainly localize in secondary lymphoid organs and circulate in the blood and are beginning to emerge as crucial players in maintaining a healthy balance between protective and pathogenic immunity (Figure 2). However, due to the localization of Tfh cells in secondary lymphoid tissues, the study of human Tfh cell heterogeneity and function in normal and disease settings has been difficult. Further analysis and comparison of human circulating counterparts to tissue-resident Tfh cells in various disease settings is critical, since it will help to reveal the level of Tfh cell heterogeneity at various stages of diseases as well as will determine whether the disease-associated alterations in Tfh cells are the main cause or result of disorders. Thus, in view of an emerging role of Tfh cells in various disease settings, we believe that current progress and further understanding of the heterogeneity and regulation of tissue-specific and cTfh cells in health and disease will lead to improved vaccine designs, better management of major autoimmune, inflammatory disorders, and cancer, and can be utilized as a novel prognostic biomarker for the identification of lymphoid and solid tumors.

## Author Contributions

All authors listed contributed significantly toward the preparation of the manuscript. LQ, TW, SB, EG, and RN wrote the manuscript. AS helped in writing, extensive editing, and revising the manuscript. HZ provided critical comments on the manuscript.

## Conflict of Interest Statement

The authors declare that the research was conducted in the absence of any commercial or financial relationships that could be construed as a potential conflict of interest.

## References

[1] Vinuesa CG Linterman MA Yu D MacLennan IC. Follicular helper T cells. Annu Rev Immunol (2016) 34:335–68. 10.1146/annurev-immunol-041015-055605 26907215

[2] Wali S Sahoo A Puri S Alekseev A Nurieva R. Insights into the development and regulation of T follicular helper cells. Cytokine (2016) 87:9–19. 10.1016/j.cyto.2016.06.010 27339151 PMC5108526

[3] Crotty S. Follicular helper CD4 T cells (TFH). Annu Rev Immunol (2011) 29:621–63. 10.1146/annurev-immunol-031210-101400 21314428

[4] Mesquita D Jr Cruvinel WM Resende LS Mesquita FV Silva NP Camara NO Follicular helper T cell in immunity and autoimmunity. Braz J Med Biol Res (2016) 49:e5209. 10.1590/1414-431X20165209 27096200 PMC4843212

[5] Karnowski A Chevrier S Belz GT Mount A Emslie D D’Costa K B and T cells collaborate in antiviral responses via IL-6, IL-21, and transcriptional activator and coactivator, Oct2 and OBF-1. J Exp Med (2012) 209:2049–64. 10.1084/jem.20111504 23045607 PMC3478936

[6] Nurieva RI Chung Y Hwang D Yang XO Kang HS Ma L Generation of T follicular helper cells is mediated by interleukin-21 but independent of T helper 1, 2, or 17 cell lineages. Immunity (2008) 29:138–49. 10.1016/j.immuni.2008.05.009 18599325 PMC2556461

[7] Nurieva RI Chung Y Martinez GJ Yang XO Tanaka S Matskevitch TD Bcl6 mediates the development of T follicular helper cells. Science (2009) 325:1001–5. 10.1126/science.1176676 19628815 PMC2857334

[8] Schmitt N Liu Y Bentebibel SE Munagala I Bourdery L Venuprasad K The cytokine TGF-beta co-opts signaling via STAT3-STAT4 to promote the differentiation of human TFH cells. Nat Immunol (2014) 15:856–65. 10.1038/ni.2947 25064073 PMC4183221

[9] Locci M Wu JE Arumemi F Mikulski Z Dahlberg C Miller AT Activin A programs the differentiation of human TFH cells. Nat Immunol (2016) 17:976–84. 10.1038/ni.3494 27376469 PMC4955732

[10] Ballesteros-Tato A Leon B Graf BA Moquin A Adams PS Lund FE Interleukin-2 inhibits germinal center formation by limiting T follicular helper cell differentiation. Immunity (2012) 36:847–56. 10.1016/j.immuni.2012.02.012 22464171 PMC3361521

[11] McDonald PW Read KA Baker CE Anderson AE Powell MD Ballesteros-Tato A IL-7 signalling represses Bcl-6 and the TFH gene program. Nat Commun (2016) 7:10285. 10.1038/ncomms10285 26743592 PMC4729877

[12] Lages CS Lewkowich I Sproles A Wills-Karp M Chougnet C Partial restoration of T-cell function in aged mice by in vitro blockade of the PD-1/PD-L1 pathway. Aging Cell (2010) 9:785–98. 10.1111/j.1474-9726.2010.00611.x 20653631 PMC2941565

[13] Sage PT Paterson AM Lovitch SB Sharpe AH. The coinhibitory receptor CTLA-4 controls B cell responses by modulating T follicular helper, T follicular regulatory, and T regulatory cells. Immunity (2014) 41:1026–39. 10.1016/j.immuni.2014.12.005 25526313 PMC4309019

[14] Nurieva RI Podd A Chen Y Alekseev AM Yu M Qi X STAT5 protein negatively regulates T follicular helper (Tfh) cell generation and function. J Biol Chem (2012) 287:11234–9. 10.1074/jbc.M111.324046 22318729 PMC3322890

[15] Stone EL Pepper M Katayama CD Kerdiles YM Lai CY Emslie E ICOS coreceptor signaling inactivates the transcription factor FOXO1 to promote Tfh cell differentiation. Immunity (2015) 42:239–51. 10.1016/j.immuni.2015.01.017 25692700 PMC4334393

[16] Weinreich MA Takada K Skon C Reiner SL Jameson SC Hogquist KA. KLF2 transcription-factor deficiency in T cells results in unrestrained cytokine production and upregulation of bystander chemokine receptors. Immunity (2009) 31:122–30. 10.1016/j.immuni.2009.05.011 19592277 PMC2724594

[17] Kim CH Rott LS Clark-Lewis I Campbell DJ Wu L Butcher EC. Subspecialization of CXCR5+ T cells: B helper activity is focused in a germinal center-localized subset of CXCR5+ T cells. J Exp Med (2001) 193:1373–81. 10.1084/jem.193.12.1373 11413192 PMC2193300

[18] Chtanova T Tangye SG Newton R Frank N Hodge MR Rolph MS T follicular helper cells express a distinctive transcriptional profile, reflecting their role as non-Th1/Th2 effector cells that provide help for B cells. J Immunol (2004) 173:68–78. 10.4049/jimmunol.173.1.68 15210760

[19] Breitfeld D Ohl L Kremmer E Ellwart J Sallusto F Lipp M Follicular B helper T cells express CXC chemokine receptor 5, localize to B cell follicles, and support immunoglobulin production. J Exp Med (2000) 192:1545–52. 10.1084/jem.192.11.1545 11104797 PMC2193094

[20] Schaerli P Willimann K Lang AB Lipp M Loetscher P Moser B. CXC chemokine receptor 5 expression defines follicular homing T cells with B cell helper function. J Exp Med (2000) 192:1553–62. 10.1084/jem.192.11.1553 11104798 PMC2193097

[21] Ansel KM McHeyzer-Williams LJ Ngo VN McHeyzer-Williams MG Cyster JG. In vivo-activated CD4 T cells upregulate CXC chemokine receptor 5 and reprogram their response to lymphoid chemokines. J Exp Med (1999) 190:1123–34. 10.1084/jem.190.8.1123 10523610 PMC2195660

[22] Forster R Mattis AE Kremmer E Wolf E Brem G Lipp M. A putative chemokine receptor, BLR1, directs B cell migration to defined lymphoid organs and specific anatomic compartments of the spleen. Cell (1996) 87:1037–47. 10.1016/S0092-8674(00)81798-5 8978608

[23] Ma CS Deenick EK Batten M Tangye SG. The origins, function, and regulation of T follicular helper cells. J Exp Med (2012) 209:1241–53. 10.1084/jem.20120994 22753927 PMC3405510

[24] Gunn MD Ngo VN Ansel KM Ekland EH Cyster JG Williams LT A B-cell-homing chemokine made in lymphoid follicles activates Burkitt’s lymphoma receptor-1. Nature (1998) 391:799–803. 10.1038/35876 9486651

[25] Kim CH Lim HW Kim JR Rott L Hillsamer P Butcher EC. Unique gene expression program of human germinal center T helper cells. Blood (2004) 104:1952–60. 10.1182/blood-2004-03-1206 15213097

[26] Johnston RJ Poholek AC DiToro D Yusuf I Eto D Barnett B Bcl6 and Blimp-1 are reciprocal and antagonistic regulators of T follicular helper cell differentiation. Science (2009) 325:1006–10. 10.1126/science.1175870 19608860 PMC2766560

[27] Yu D Rao S Tsai LM Lee SK He Y Sutcliffe EL The transcriptional repressor Bcl-6 directs T follicular helper cell lineage commitment. Immunity (2009) 31:457–68. 10.1016/j.immuni.2009.07.002 19631565

[28] Ueno H Banchereau J Vinuesa CG. Pathophysiology of T follicular helper cells in humans and mice. Nat Immunol (2015) 16:142–52. 10.1038/ni.3054 25594465 PMC4459756

[29] Flynn S Toellner KM Raykundalia C Goodall M Lane P. CD4 T cell cytokine differentiation: the B cell activation molecule, OX40 ligand, instructs CD4 T cells to express interleukin 4 and upregulates expression of the chemokine receptor, Blr-1. J Exp Med (1998) 188:297–304. 10.1084/jem.188.2.297 9670042 PMC2212448

[30] Kerfoot SM Yaari G Patel JR Johnson KL Gonzalez DG Kleinstein SH Germinal center B cell and T follicular helper cell development initiates in the interfollicular zone. Immunity (2011) 34:947–60. 10.1016/j.immuni.2011.03.024 21636295 PMC3280079

[31] Kitano M Moriyama S Ando Y Hikida M Mori Y Kurosaki T Bcl6 protein expression shapes pre-germinal center B cell dynamics and follicular helper T cell heterogeneity. Immunity (2011) 34:961–72. 10.1016/j.immuni.2011.03.025 21636294

[32] Haynes NM Allen CD Lesley R Ansel KM Killeen N Cyster JG Role of CXCR5 and CCR7 in follicular Th cell positioning and appearance of a programmed cell death gene-1high germinal center-associated subpopulation. J Immunol (2007) 179:5099–108. 10.4049/jimmunol.179.8.5099 17911595

[33] Ramiscal RR Vinuesa CG. T-cell subsets in the germinal center. Immunol Rev (2013) 252:146–55. 10.1111/imr.12031 23405902

[34] Bentebibel SE Schmitt N Banchereau J Ueno H. Human tonsil B-cell lymphoma 6 (BCL6)-expressing CD4+ T-cell subset specialized for B-cell help outside germinal centers. Proc Natl Acad Sci U S A (2011) 108:E488–97. 10.1073/pnas.1100898108 21808017 PMC3158181

[35] Nakayamada S Kanno Y Takahashi H Jankovic D Lu KT Johnson TA Early Th1 cell differentiation is marked by a Tfh cell-like transition. Immunity (2011) 35:919–31. 10.1016/j.immuni.2011.11.012 22195747 PMC3244883

[36] Bauquet AT Jin H Paterson AM Mitsdoerffer M Ho IC Sharpe AH The costimulatory molecule ICOS regulates the expression of c-Maf and IL-21 in the development of follicular T helper cells and TH-17 cells. Nat Immunol (2009) 10:167–75. 10.1038/ni.1690 19098919 PMC2742982

[37] He J Tsai LM Leong YA Hu X Ma CS Chevalier N Circulating precursor CCR7(lo)PD-1(hi) CXCR5(+) CD4(+) T cells indicate Tfh cell activity and promote antibody responses upon antigen reexposure. Immunity (2013) 39:770–81. 10.1016/j.immuni.2013.09.007 24138884

[38] Morita R Schmitt N Bentebibel SE Ranganathan R Bourdery L Zurawski G Human blood CXCR5(+)CD4(+) T cells are counterparts of T follicular cells and contain specific subsets that differentially support antibody secretion. Immunity (2011) 34:108–21. 10.1016/j.immuni.2010.12.012 21215658 PMC3046815

[39] Schmitt N Bentebibel SE Ueno H. Phenotype and functions of memory Tfh cells in human blood. Trends Immunol (2014) 35:436–42. 10.1016/j.it.2014.06.002 24998903 PMC4152409

[40] Asrir A Aloulou M Gador M Perals C Fazilleau N. Interconnected subsets of memory follicular helper T cells have different effector functions. Nat Commun (2017) 8:847. 10.1038/s41467-017-00843-7 29018187 PMC5635037

[41] Scherm MG Ott VB Daniel C. Follicular helper T cells in autoimmunity. Curr Diab Rep (2016) 16:75. 10.1007/s11892-016-0770-2 27324759

[42] Schmitt N Ueno H. Regulation of human helper T cell subset differentiation by cytokines. Curr Opin Immunol (2015) 34:130–6. 10.1016/j.coi.2015.03.007 25879814 PMC4465198

[43] Weinstein JS Hernandez SG Craft J. T cells that promote B-cell maturation in systemic autoimmunity. Immunol Rev (2012) 247:160–71. 10.1111/j.1600-065X.2012.01122.x 22500839 PMC3334351

[44] Jogdand GM Mohanty S Devadas S. Regulators of Tfh cell differentiation. Front Immunol (2016) 7:520. 10.3389/fimmu.2016.00520 27933060 PMC5120123

[45] Yang JH Zhang J Cai Q Zhao DB Wang J Guo PE Expression and function of inducible costimulator on peripheral blood T cells in patients with systemic lupus erythematosus. Rheumatology (Oxford) (2005) 44:1245–54. 10.1093/rheumatology/keh724 15987711

[46] Gong F Qian C Zhu H Zhu J Pan Y Dong Q Circulating follicular T-helper cell subset distribution in patients with asthma. Allergy Asthma Proc (2016) 37:154–61. 10.2500/aap.2016.37.3982 27931292

[47] Weber JP Fuhrmann F Feist RK Lahmann A Al Baz MS Gentz LJ ICOS maintains the T follicular helper cell phenotype by down-regulating Kruppel-like factor 2. J Exp Med (2015) 212:217–33. 10.1084/jem.20141432 25646266 PMC4322049

[48] Alegre ML Frauwirth KA Thompson CB. T-cell regulation by CD28 and CTLA-4. Nat Rev Immunol (2001) 1:220–8. 10.1038/35105024 11905831

[49] Peach RJ Bajorath J Brady W Leytze G Greene J Naemura J Complementarity determining region 1 (CDR1)- and CDR3-analogous regions in CTLA-4 and CD28 determine the binding to B7-1. J Exp Med (1994) 180(6):2049–58. 10.1084/jem.180.6.2049 7964482 PMC2191786

[50] Bluestone J New perspectives of CD28-B7-mediated T cell costimulation. Immunity (1995) 2(6):555–9. 10.1016/1074-7613(95)90000-4 7540940

[51] Salek-Ardakani S Choi YS Rafii-El-Idrissi Benhnia M Flynn R Arens R Shoenberger S B cell-specific expression of B7-2 is required for follicular Th cell function in response to vaccinia virus. J Immunol (2011) 186:5294–303. 10.4049/jimmunol.1100406 21441451 PMC3089765

[52] Wang CJ Heuts F Ovcinnikovs V Wardzinski L Bowers C Schmidt EM CTLA-4 controls follicular helper T-cell differentiation by regulating the strength of CD28 engagement. Proc Natl Acad Sci U S A (2015) 112:524–9. 10.1073/pnas.1414576112 25548162 PMC4299196

[53] Linterman MA Denton AE Divekar DP Zvetkova I Kane L Ferreira C CD28 expression is required after T cell priming for helper T cell responses and protective immunity to infection. Elife (2014) 3:e03180. 10.7554/eLife.03180 25347065 PMC4241536

[54] Leavenworth JW Verbinnen B Yin J Huang H Cantor H A p85alpha-osteopontin axis couples the receptor ICOS to sustained Bcl-6 expression by follicular helper and regulatory T cells. Nat Immunol (2015) 16:96–106. 10.1038/ni.3050 25436971 PMC4405167

[55] Chen S Cai C Li Z Liu G Wang Y Blonska M Dissection of SAP-dependent and SAP-independent SLAM family signaling in NKT cell development and humoral immunity. J Exp Med (2017) 214:475–89. 10.1084/jem.20161312 28049627 PMC5294859

[56] Ma CS Deenick EK. The role of SAP and SLAM family molecules in the humoral immune response. Ann N Y Acad Sci (2011) 1217:32–44. 10.1111/j.1749-6632.2010.05824.x 21091715

[57] Cannons JL Tangye SG Schwartzberg PL. SLAM family receptors and SAP adaptors in immunity. Annu Rev Immunol (2011) 29:665–705. 10.1146/annurev-immunol-030409-101302 21219180

[58] Cannons JL Qi H Lu KT Dutta M Gomez-Rodriguez J Cheng J Optimal germinal center responses require a multistage T cell:B cell adhesion process involving integrins, SLAM-associated protein, and CD84. Immunity (2010) 32:253–65. 10.1016/j.immuni.2010.01.010 20153220 PMC2830297

[59] Kageyama R Cannons JL Zhao F Yusuf I Lao C Locci M The receptor Ly108 functions as a SAP adaptor-dependent on-off switch for T cell help to B cells and NKT cell development. Immunity (2012) 36:986–1002. 10.1016/j.immuni.2012.05.016 22683125 PMC3389310

[60] Yusuf I Kageyama R Monticelli L Johnston RJ Ditoro D Hansen K Germinal center T follicular helper cell IL-4 production is dependent on signaling lymphocytic activation molecule receptor (CD150). J Immunol (2010) 185:190–202. 10.4049/jimmunol.0903505 20525889 PMC2913439

[61] Crotty S Kersh EN Cannons J Schwartzberg PL Ahmed R. SAP is required for generating long-term humoral immunity. Nature (2003) 421:282–7. 10.1038/nature01318 12529646

[62] Kroenke MA Eto D Locci M Cho M Davidson T Haddad EK Bcl6 and Maf cooperate to instruct human follicular helper CD4 T cell differentiation. J Immunol (2012) 188:3734–44. 10.4049/jimmunol.1103246 22427637 PMC3324673

[63] Hu J Havenar-Daughton C Crotty S. Modulation of SAP dependent T:B cell interactions as a strategy to improve vaccination. Curr Opin Virol (2013) 3:363–70. 10.1016/j.coviro.2013.05.015 23743125 PMC3763741

[64] Cannons JL Yu LJ Jankovic D Crotty S Horai R Kirby M SAP regulates T cell-mediated help for humoral immunity by a mechanism distinct from cytokine regulation. J Exp Med (2006) 203:1551–65. 10.1084/jem.20052097 16754717 PMC2118305

[65] Qi H Cannons JL Klauschen F Schwartzberg PL Germain RN. SAP-controlled T-B cell interactions underlie germinal centre formation. Nature (2008) 455:764–9. 10.1038/nature07345 18843362 PMC2652134

[66] Latour S Roncagalli R Chen R Bakinowski M Shi X Schwartzberg PL Binding of SAP SH2 domain to FynT SH3 domain reveals a novel mechanism of receptor signalling in immune regulation. Nat Cell Biol (2003) 5:149–54. 10.1038/ncb919 12545173

[67] Cannons JL Wu JZ Gomez-Rodriguez J Zhang J Dong B Liu Y Biochemical and genetic evidence for a SAP-PKC-theta interaction contributing to IL-4 regulation. J Immunol (2010) 185:2819–27. 10.4049/jimmunol.0902182 20668219 PMC3422635

[68] Zhao F Cannons JL Dutta M Griffiths GM Schwartzberg PL. Positive and negative signaling through SLAM receptors regulate synapse organization and thresholds of cytolysis. Immunity (2012) 36:1003–16. 10.1016/j.immuni.2012.05.017 22683123 PMC3389133

[69] Nocentini G Giunchi L Ronchetti S Krausz LT Bartoli A Moraca R A new member of the tumor necrosis factor/nerve growth factor receptor family inhibits T cell receptor-induced apoptosis. Proc Nat Acad Sci U S A (1997) 94:6216–21. 10.1073/pnas.94.12.6216 9177197 PMC21029

[70] Knee DA Hewes B Brogdon JL. Rationale for anti-GITR cancer immunotherapy. Eur J Cancer (2016) 67:1–10. 10.1016/j.ejca.2016.06.028 27591414

[71] Stephens GL McHugh RS Whitters MJ Young DA Luxenberg D Carreno BM Engagement of glucocorticoid-induced TNFR family-related receptor on effector T cells by its ligand mediates resistance to suppression by CD4+CD25+ T cells. J Immunol (2004) 173:5008–20. 10.4049/jimmunol.173.8.5008 15470044

[72] Butcher MJ Filipowicz AR Waseem TC McGary CM Crow KJ Magilnick N Atherosclerosis-driven Treg plasticity results in formation of a dysfunctional subset of plastic IFNgamma+ Th1/Tregs. Circ Res (2016) 119:1190–203. 10.1161/CIRCRESAHA.116.309764 27635087 PMC5242312

[73] Clouthier DL Zhou AC Wortzman ME Luft O Levy GA Watts TH. GITR intrinsically sustains early type 1 and late follicular helper CD4 T cell accumulation to control a chronic viral infection. PLoS Pathog (2015) 11:e1004517. 10.1371/journal.ppat.1004517 25590581 PMC4295864

[74] Good-Jacobson KL Szumilas CG Chen L Sharpe AH Tomayko MM Shlomchik MJ. PD-1 regulates germinal center B cell survival and the formation and affinity of long-lived plasma cells. Nat Immunol (2010) 11:535–42. 10.1038/ni.1877 20453843 PMC2874069

[75] Chung Y Tanaka S Chu F Nurieva RI Martinez GJ Rawal S Follicular regulatory T cells expressing Foxp3 and Bcl-6 suppress germinal center reactions. Nat Med (2011) 17:983–8. 10.1038/nm.2426 21785430 PMC3151340

[76] Linterman MA Pierson W Lee SK Kallies A Kawamoto S Rayner TF Foxp3+ follicular regulatory T cells control the germinal center response. Nat Med (2011) 17:975–82. 10.1038/nm.2425 21785433 PMC3182542

[77] Sage PT Francisco LM Carman CV Sharpe AH. The receptor PD-1 controls follicular regulatory T cells in the lymph nodes and blood. Nat Immunol (2013) 14:152–61. 10.1038/ni.2496 23242415 PMC3788614

[78] Sage PT Sharpe AH. T follicular regulatory cells. Immunol Rev (2016) 271:246–59. 10.1111/imr.12411 27088919

[79] Francisco LM Sage PT Sharpe AH. The PD-1 pathway in tolerance and autoimmunity. Immunol Rev (2010) 236:219–42. 10.1111/j.1600-065X.2010.00923.x 20636820 PMC2919275

[80] Keir ME Butte MJ Freeman GJ Sharpe AH. PD-1 and its ligands in tolerance and immunity. Annu Rev Immunol (2008) 26:677–704. 10.1146/annurev.immunol.26.021607.090331 18173375 PMC10637733

[81] Hamel KM Cao Y Wang Y Rodeghero R Kobezda T Chen L B7-H1 expression on non-B and non-T cells promotes distinct effects on T- and B-cell responses in autoimmune arthritis. Eur J Immunol (2010) 40:3117–27. 10.1002/eji.201040690 21061440 PMC3638795

[82] Kawamoto S Tran TH Maruya M Suzuki K Doi Y Tsutsui Y The inhibitory receptor PD-1 regulates IgA selection and bacterial composition in the gut. Science (2012) 336:485–9. 10.1126/science.1217718 22539724

[83] Hams E McCarron MJ Amu S Yagita H Azuma M Chen L Blockade of B7-H1 (programmed death ligand 1) enhances humoral immunity by positively regulating the generation of T follicular helper cells. J Immunol (2011) 186:5648–55. 10.4049/jimmunol.1003161 21490158

[84] Velu V Titanji K Zhu B Husain S Pladevega A Lai L Enhancing SIV-specific immunity in vivo by PD-1 blockade. Nature (2009) 458:206–10. 10.1038/nature07662 19078956 PMC2753387

[85] Butler NS Moebius J Pewe LL Traore B Doumbo OK Tygrett LT Therapeutic blockade of PD-L1 and LAG-3 rapidly clears established blood-stage Plasmodium infection. Nat Immunol (2011) 13:188–95. 10.1038/ni.2180 22157630 PMC3262959

[86] Sage PT Tan CL Freeman GJ Haigis M Sharpe AH. Defective TFH cell function and increased TFR cells contribute to defective antibody production in aging. Cell Rep (2015) 12:163–71. 10.1016/j.celrep.2015.06.015 26146074 PMC4504745

[87] Badell IR La Muraglia GM II Liu D Wagener ME Ding G Ford ML. Selective CD28 blockade results in superior inhibition of donor-specific T follicular helper cell and antibody responses relative to CTLA4-Ig. Am J Transplant (2018) 18:89–101. 10.1111/ajt.14400 28637095 PMC5740006

[88] Walunas TL Lenschow DJ Bakker CY Linsley PS Freeman GJ Green JM CTLA-4 can function as a negative regulator of T cell activation. Immunity (1994) 1:405–13. 10.1016/1074-7613(94)90071-X 7882171

[89] Krummel MF. CD28 and CTLA-4 have opposing effects on the response of T cells to stimulation. J Exp Med (1995) 182:459–65. 10.1084/jem.182.2.459 7543139 PMC2192127

[90] Tivol EA Borriello F Schweitzer AN Lynch WP Bluestone JA Sharpe AH. Loss of CTLA-4 leads to massive lymphoproliferation and fatal multiorgan tissue destruction, revealing a critical negative regulatory role of CTLA-4. Immunity (1995) 3:541–7. 10.1016/1074-7613(95)90125-6 7584144

[91] Perez VL Van Parijs L Biuckians A Zheng XX Strom TB Abbas AK. Induction of peripheral T cell tolerance in vivo requires CTLA-4 engagement. Immunity (1997) 6:411–7. 10.1016/S1074-7613(00)80284-8 9133420

[92] Leach DR Krummel MF Allison JP. Enhancement of antitumor immunity by CTLA-4 blockade. Science (1996) 271:1734–6. 10.1126/science.271.5256.1734 8596936

[93] Walker LS. Treg and CTLA-4: two intertwining pathways to immune tolerance. J Autoimmun (2013) 45:49–57. 10.1016/j.jaut.2013.06.006 23849743 PMC3989116

[94] Tivol EA Boyd SD McKeon S Borriello F Nickerson P Strom TB CTLA4Ig prevents lymphoproliferation and fatal multiorgan tissue destruction in CTLA-4-deficient mice. J Immunol (1997) 158:5091–4. 9164923

[95] Tang Q Boden EK Henriksen KJ Bour-Jordan H Bi M Bluestone JA. Distinct roles of CTLA-4 and TGF-beta in CD4+CD25+ regulatory T cell function. Eur J Immunol (2004) 34:2996–3005. 10.1002/eji.200425143 15468055

[96] Wing K Onishi Y Prieto-Martin P Yamaguchi T Miyara M Fehervari Z CTLA-4 control over Foxp3+ regulatory T cell function. Science (2008) 322:271–5. 10.1126/science.1160062 18845758

[97] Wing JB Ise W Kurosaki T Sakaguchi S. Regulatory T cells control antigen-specific expansion of Tfh cell number and humoral immune responses via the coreceptor CTLA-4. Immunity (2014) 41:1013–25. 10.1016/j.immuni.2014.12.006 25526312

[98] Cheung TC Humphreys IR Potter KG Norris PS Shumway HM Tran BR Evolutionarily divergent herpesviruses modulate T cell activation by targeting the herpesvirus entry mediator cosignaling pathway. Proc Natl Acad Sci U S A (2005) 102:13218–23. 10.1073/pnas.0506172102 16131544 PMC1201609

[99] del Rio ML Jones ND Buhler L Norris P Shintani Y Ware CF Selective blockade of herpesvirus entry mediator-B and T lymphocyte attenuator pathway ameliorates acute graft-versus-host reaction. J Immunol (2012) 188:4885–96. 10.4049/jimmunol.1103698 22490863 PMC3925259

[100] del Rio ML Kaye J Rodriguez-Barbosa JI. Detection of protein on BTLAlow cells and in vivo antibody-mediated down-modulation of BTLA on lymphoid and myeloid cells of C57BL/6 and BALB/c BTLA allelic variants. Immunobiology (2010) 215:570–8. 10.1016/j.imbio.2009.09.008 19837478

[101] Oya Y Watanabe N Owada T Oki M Hirose K Suto A Development of autoimmune hepatitis-like disease and production of autoantibodies to nuclear antigens in mice lacking B and T lymphocyte attenuator. Arthritis Rheum (2008) 58:2498–510. 10.1002/art.23674 18668554 PMC2782777

[102] Kashiwakuma D Suto A Hiramatsu Y Ikeda K Takatori H Suzuki K B and T lymphocyte attenuator suppresses IL-21 production from follicular Th cells and subsequent humoral immune responses. J Immunol (2010) 185:2730–6. 10.4049/jimmunol.0903839 20660710

[103] Eto D Lao C DiToro D Barnett B Escobar TC Kageyama R IL-21 and IL-6 are critical for different aspects of B cell immunity and redundantly induce optimal follicular helper CD4 T cell (Tfh) differentiation. PLoS One (2011) 6:e17739 10.1371/journal.pone.0017739 21423809 PMC3056724

[104] Batten M Ramamoorthi N Kljavin NM Ma CS Cox JH Dengler HS IL-27 supports germinal center function by enhancing IL-21 production and the function of T follicular helper cells. J Exp Med (2010) 207:2895–906. 10.1084/jem.20100064 21098093 PMC3005229

[105] Nurieva RI Chung Y. Understanding the development and function of T follicular helper cells. Cell Mol Immunol (2010) 7:190–7. 10.1038/cmi.2010.24 20383172 PMC4002918

[106] Liu X Nurieva RI Dong C. Transcriptional regulation of follicular T-helper (Tfh) cells. Immunol Rev (2013) 252:139–45. 10.1111/imr.12040 23405901 PMC3579502

[107] Choi YS Eto D Yang JA Lao C Crotty S Cutting edge: STAT1 is required for IL-6-mediated Bcl6 induction for early follicular helper cell differentiation. J Immunol (2013) 190:3049–53. 10.4049/jimmunol.1203032 23447690 PMC3626564

[108] Chavele KM Merry E Ehrenstein MR. Cutting edge: circulating plasmablasts induce the differentiation of human T follicular helper cells via IL-6 production. J Immunol (2015) 194:2482–5. 10.4049/jimmunol.1401190 25681343 PMC4356730

[109] Nurieva R Yang XO Martinez G Zhang Y Panopoulos AD Ma L Essential autocrine regulation by IL-21 in the generation of inflammatory T cells. Nature (2007) 448:480–3. 10.1038/nature05969 17581589

[110] Vogelzang A McGuire HM Yu D Sprent J Mackay CR King C. A fundamental role for interleukin-21 in the generation of T follicular helper cells. Immunity (2008) 29:127–37. 10.1016/j.immuni.2008.06.001 18602282

[111] Gringhuis SI Kaptein TM Wevers BA van der Vlist M Klaver EJ van Die I Fucose-based PAMPs prime dendritic cells for follicular T helper cell polarization via DC-SIGN-dependent IL-27 production. Nat Commun (2014) 5:5074. 10.1038/ncomms6074 25278262

[112] Owaki T Asakawa M Kamiya S Takeda K Fukai F Mizuguchi J IL-27 suppresses CD28-mediated [correction of medicated] IL-2 production through suppressor of cytokine signaling 3. J Immunol (2006) 176:2773–80. 10.4049/jimmunol.176.5.2773 16493033

[113] Peng X Remacle JE Kasran A Huylebroeck D Ceuppens JL. IL-12 up-regulates CD40 ligand (CD154) expression on human T cells. J Immunol (1998) 160:1166–72. 9570530

[114] McCarron MJ Marie JC TGF-beta prevents T follicular helper cell accumulation and B cell autoreactivity. J Clin Invest (2014) 124:4375–86. 10.1172/JCI76179 25157822 PMC4191003

[115] Choi YS Kageyama R Eto D Escobar TC Johnston RJ Monticelli L ICOS receptor instructs T follicular helper cell versus effector cell differentiation via induction of the transcriptional repressor Bcl6. Immunity (2011) 34:932–46. 10.1016/j.immuni.2011.03.023 21636296 PMC3124577

[116] Pepper M Pagan AJ Igyarto BZ Taylor JJ Jenkins MK. Opposing signals from the Bcl6 transcription factor and the interleukin-2 receptor generate T helper 1 central and effector memory cells. Immunity (2011) 35:583–95. 10.1016/j.immuni.2011.09.009 22018468 PMC3208313

[117] Liao W Lin JX Wang L Li P Leonard WJ. Modulation of cytokine receptors by IL-2 broadly regulates differentiation into helper T cell lineages. Nat Immunol (2011) 12:551–9. 10.1038/ni.2030 21516110 PMC3304099

[118] Ray JP Staron MM Shyer JA Ho PC Marshall HD Gray SM The interleukin-2-mTORc1 kinase axis defines the signaling, differentiation, and metabolism of T helper 1 and follicular B Helper T cells. Immunity (2015) 43:690–702. 10.1016/j.immuni.2015.08.017 26410627 PMC4618086

[119] Cai G Nie X Zhang W Wu B Lin J Wang H A regulatory role for IL-10 receptor signaling in development and B cell help of T follicular helper cells in mice. J Immunol (2012) 189:1294–302. 10.4049/jimmunol.1102948 22753938

[120] Sahoo A Alekseev A Tanaka K Obertas L Lerman B Haymaker C Batf is important for IL-4 expression in T follicular helper cells. Nat Commun (2015) 6:7997. 10.1038/ncomms8997 26278622 PMC4557271

[121] Gong F Su Q Jiang D Chen J Pan Y Huang X. High frequency of circulating follicular helper T cells in patients with bronchial asthma. Clin Lab (2014) 60:963–8. 10.7754/Clin.Lab.2013.130427 25016701

[122] Crotty S Johnston RJ Schoenberger SP. Effectors and memories: Bcl-6 and Blimp-1 in T and B lymphocyte differentiation. Nat Immunol (2010) 11:114–20. 10.1038/ni.1837 20084069 PMC2864556

[123] Martins G Calame K. Regulation and functions of Blimp-1 in T and B lymphocytes. Annu Rev Immunol (2008) 26:133–69. 10.1146/annurev.immunol.26.021607.090241 18370921

[124] Hatzi K Nance JP Kroenke MA Bothwell M Haddad EK Melnick A BCL6 orchestrates Tfh cell differentiation via multiple distinct mechanisms. J Exp Med (2015) 212:539–53. 10.1084/jem.20141380 25824819 PMC4387288

[125] Kusam S Toney LM Sato H Dent AL Inhibition of Th2 differentiation and GATA-3 expression by BCL-6. J Immunol (2003) 170:2435–41. 10.4049/jimmunol.170.5.2435 12594267

[126] Liu X Lu H Chen T Nallaparaju KC Yan X Tanaka S Genome-wide analysis identifies Bcl6-controlled regulatory networks during T follicular helper cell differentiation. Cell Rep (2016) 14:1735–47. 10.1016/j.celrep.2016.01.038 26876184 PMC4975778

[127] Andris F Denanglaire S Anciaux M Hercor M Hussein H Leo O. The transcription factor c-Maf promotes the differentiation of follicular helper T cells. Front Immunol (2017) 8:480. 10.3389/fimmu.2017.00480 28496444 PMC5406410

[128] Schraml BU Hildner K Ise W Lee WL Smith WA Solomon B The AP-1 transcription factor Batf controls T(H)17 differentiation. Nature (2009) 460:405–9. 10.1038/nature08114 19578362 PMC2716014

[129] Betz BC Jordan-Williams KL Wang C Kang SG Liao J Logan MR Batf coordinates multiple aspects of B and T cell function required for normal antibody responses. J Exp Med (2010) 207:933–42. 10.1084/jem.20091548 20421391 PMC2867277

[130] Ise W Kohyama M Schraml BU Zhang T Schwer B Basu U The transcription factor BATF controls the global regulators of class-switch recombination in both B cells and T cells. Nat Immunol (2011) 12:536–43. 10.1038/ni.2037 21572431 PMC3117275

[131] Brustle A Heink S Huber M Rosenplanter C Stadelmann C Yu P The development of inflammatory T(H)-17 cells requires interferon-regulatory factor 4. Nat Immunol (2007) 8:958–66. 10.1038/ni1500 17676043

[132] Huber M Brustle A Reinhard K Guralnik A Walter G Mahiny A IRF4 is essential for IL-21-mediated induction, amplification, and stabilization of the Th17 phenotype. Proc Natl Acad Sci U S A (2008) 105:20846–51. 10.1073/pnas.0809077106 19088203 PMC2634912

[133] Lohoff M Mittrucker HW Prechtl S Bischof S Sommer F Kock S Dysregulated T helper cell differentiation in the absence of interferon regulatory factor 4. Proc Natl Acad Sci U S A (2002) 99:11808–12. 10.1073/pnas.182425099 12189207 PMC129350

[134] Rengarajan J Mowen KA McBride KD Smith ED Singh H Glimcher LH. Interferon regulatory factor 4 (IRF4) interacts with NFATc2 to modulate interleukin 4 gene expression. J Exp Med (2002) 195:1003–12. 10.1084/jem.20011128 11956291 PMC2193700

[135] Zheng Y Chaudhry A Kas A deRoos P Kim JM Chu TT Regulatory T-cell suppressor program co-opts transcription factor IRF4 to control T(H)2 responses. Nature (2009) 458:351–6. 10.1038/nature07674 19182775 PMC2864791

[136] Chen Q Yang W Gupta S Biswas P Smith P Bhagat G IRF-4-binding protein inhibits interleukin-17 and interleukin-21 production by controlling the activity of IRF-4 transcription factor. Immunity (2008) 29:899–911. 10.1016/j.immuni.2008.10.011 19062315 PMC2633410

[137] Staudt V Bothur E Klein M Lingnau K Reuter S Grebe N Interferon-regulatory factor 4 is essential for the developmental program of T helper 9 cells. Immunity (2010) 33:192–202. 10.1016/j.immuni.2010.07.014 20674401

[138] Klein U Casola S Cattoretti G Shen Q Lia M Mo T Transcription factor IRF4 controls plasma cell differentiation and class-switch recombination. Nat Immunol (2006) 7:773–82. 10.1038/ni1357 16767092

[139] Sciammas R Shaffer AL Schatz JH Zhao H Staudt LM Singh H. Graded expression of interferon regulatory factor-4 coordinates isotype switching with plasma cell differentiation. Immunity (2006) 25:225–36. 10.1016/j.immuni.2006.07.009 16919487

[140] Bollig N Brustle A Kellner K Ackermann W Abass E Raifer H Transcription factor IRF4 determines germinal center formation through follicular T-helper cell differentiation. Proc Natl Acad Sci U S A (2012) 109:8664–9. 10.1073/pnas.1205834109 22552227 PMC3365194

[141] Li P Spolski R Liao W Wang L Murphy TL Murphy KM BATF-JUN is critical for IRF4-mediated transcription in T cells. Nature (2012) 490:543–6. 10.1038/nature11530 22992523 PMC3537508

[142] Kwon H Thierry-Mieg D Thierry-Mieg J Kim HP Oh J Tunyaplin C Analysis of interleukin-21-induced Prdm1 gene regulation reveals functional cooperation of STAT3 and IRF4 transcription factors. Immunity (2009) 31:941–52. 10.1016/j.immuni.2009.10.008 20064451 PMC3272079

[143] Gupta S Jiang M Anthony A Pernis AB. Lineage-specific modulation of interleukin 4 signaling by interferon regulatory factor 4. J Exp Med (1999) 190:1837–48. 10.1084/jem.190.12.1837 10601358 PMC2195723

[144] Krishnamoorthy V Kannanganat S Maienschein-Cline M Cook SL Chen J Bahroos N The IRF4 gene regulatory module functions as a read-write integrator to dynamically coordinate T helper cell fate. Immunity (2017) 47:481–97.e7. 10.1016/j.immuni.2017.09.001 28930660 PMC5661949

[145] Nakayamada S Poholek AC Lu KT Takahashi H Kato M Iwata S Type I IFN induces binding of STAT1 to Bcl6: divergent roles of STAT family transcription factors in the T follicular helper cell genetic program. J Immunol (2014) 192:2156–66. 10.4049/jimmunol.1300675 24489092 PMC3967131

[146] Zhong Z Wen Z Darnell JE Jr. Stat3: a STAT family member activated by tyrosine phosphorylation in response to epidermal growth factor and interleukin-6. Science (1994) 264:95–8. 10.1126/science.8140422 8140422

[147] Asao H Okuyama C Kumaki S Ishii N Tsuchiya S Foster D Cutting edge: the common gamma-chain is an indispensable subunit of the IL-21 receptor complex. J Immunol (2001) 167:1–5. 10.4049/jimmunol.167.1.1 11418623

[148] Read KA Powell MD Baker CE Sreekumar BK Ringel-Scaia VM Bachus H Integrated STAT3 and Ikaros zinc finger transcription factor activities regulate Bcl-6 expression in CD4(+) Th cells. J Immunol (2017) 199:2377–87. 10.4049/jimmunol.1700106 28848064 PMC5657606

[149] Ma CS Avery DT Chan A Batten M Bustamante J Boisson-Dupuis S Functional STAT3 deficiency compromises the generation of human T follicular helper cells. Blood (2012) 119:3997–4008. 10.1182/blood-2011-11-392985 22403255 PMC3355712

[150] Schmitt N Morita R Bourdery L Bentebibel SE Zurawski SM Banchereau J Human dendritic cells induce the differentiation of interleukin-21-producing T follicular helper-like cells through interleukin-12. Immunity (2009) 31:158–69. 10.1016/j.immuni.2009.04.016 19592276 PMC2731623

[151] Weinstein JS Laidlaw BJ Lu Y Wang JK Schulz VP Li N STAT4 and T-bet control follicular helper T cell development in viral infections. J Exp Med (2018) 215:337–55. 10.1084/jem.2017045702062018c 29212666 PMC5748849

[152] Auderset F Coutaz M Tacchini-Cottier F The role of Notch in the differentiation of CD4(+) T helper cells. Curr Top Microbiol Immunol (2012) 360:115–34. 10.1007/82_2012_227 22653552

[153] Auderset F Schuster S Fasnacht N Coutaz M Charmoy M Koch U Notch signaling regulates follicular helper T cell differentiation. J Immunol (2013) 191:2344–50. 10.4049/jimmunol.1300643 23918982

[154] Dell’Aringa M Reinhardt RL. Notch signaling represents an important checkpoint between follicular T-helper and canonical T-helper 2 cell fate. Mucosal Immunol (2018) 11:1079–91. 10.1038/s41385-018-0012-9 29467447 PMC6030499

[155] Vaeth M Muller G Stauss D Dietz L Klein-Hessling S Serfling E Follicular regulatory T cells control humoral autoimmunity via NFAT2-regulated CXCR5 expression. J Exp Med (2014) 211:545–61. 10.1084/jem.20130604 24590764 PMC3949566

[156] Martinez GJ Hu JK Pereira RM Crampton JS Togher S Bild N Cutting edge: NFAT transcription factors promote the generation of follicular helper T cells in response to acute viral infection. J Immunol (2016) 196:2015–9. 10.4049/jimmunol.1501841 26851216 PMC4761453

[157] Liu X Chen X Zhong B Wang A Wang X Chu F Transcription factor achaete-scute homologue 2 initiates follicular T-helper-cell development. Nature (2014) 507:513–8. 10.1038/nature12910 24463518 PMC4012617

[158] Choi YS Gullicksrud JA Xing S Zeng Z Shan Q Li F LEF-1 and TCF-1 orchestrate T(FH) differentiation by regulating differentiation circuits upstream of the transcriptional repressor Bcl6. Nat Immunol (2015) 16:980–90. 10.1038/ni.3226 26214741 PMC4545301

[159] Wu T Shin HM Moseman EA Ji Y Huang B Harly C TCF1 is required for the T follicular helper cell response to viral infection. Cell Rep (2015) 12:2099–110. 10.1016/j.celrep.2015.08.049 26365183 PMC4591235

[160] Xu L Cao Y Xie Z Huang Q Bai Q Yang X The transcription factor TCF-1 initiates the differentiation of T(FH) cells during acute viral infection. Nat Immunol (2015) 16:991–9. 10.1038/ni.3229 26214740

[161] Weber BN Chi AW Chavez A Yashiro-Ohtani Y Yang Q Shestova O A critical role for TCF-1 in T-lineage specification and differentiation. Nature (2011) 476:63–8. 10.1038/nature10279 21814277 PMC3156435

[162] Yu S Zhou X Steinke FC Liu C Chen SC Zagorodna O The TCF-1 and LEF-1 transcription factors have cooperative and opposing roles in T cell development and malignancy. Immunity (2012) 37:813–26. 10.1016/j.immuni.2012.08.009 23103132 PMC3501598

[163] Ogbe A Miao T Symonds AL Omodho B Singh R Bhullar P Early growth response genes 2 and 3 regulate the expression of Bcl6 and differentiation of T follicular helper cells. J Biol Chem (2015) 290:20455–65. 10.1074/jbc.M114.634816 25979336 PMC4536451

[164] Stauss D Brunner C Berberich-Siebelt F Hopken UE Lipp M Muller G. The transcriptional coactivator Bob1 promotes the development of follicular T helper cells via Bcl6. EMBO J (2016) 35:881–98. 10.15252/embj.201591459 26957522 PMC4972135

[165] Barnett LG Simkins HM Barnett BE Korn LL Johnson AL Wherry EJ B cell antigen presentation in the initiation of follicular helper T cell and germinal center differentiation. J Immunol (2014) 192:3607–17. 10.4049/jimmunol.1301284 24646739 PMC4380085

[166] Schwickert TA Victora GD Fooksman DR Kamphorst AO Mugnier MR Gitlin AD T cell-limited checkpoint regulates affinity-dependent B cell entry into the germinal center. J Exp Med (2011) 208:1243–52. 10.1084/jem.20102477 21576382 PMC3173244

[167] Serre K Mohr E Benezech C Bird R Khan M Caamano JH Selective effects of NF-kappaB1 deficiency in CD4(+) T cells on Th2 and TFh induction by alum-precipitated protein vaccines. Eur J Immunol (2011) 41:1573–82. 10.1002/eji.201041126 21469117

[168] Hu H Wu X Jin W Chang M Cheng X Sun SC. Noncanonical NF-kappaB regulates inducible costimulator (ICOS) ligand expression and T follicular helper cell development. Proc Natl Acad Sci U S A (2011) 108:12827–32. 10.1073/pnas.1105774108 21768353 PMC3150902

[169] Xiao N Eto D Elly C Peng G Crotty S Liu YC. The E3 ubiquitin ligase Itch is required for the differentiation of follicular helper T cells. Nat Immunol (2014) 15:657–66. 10.1038/ni.2912 24859451 PMC4289613

[170] Wang H Geng J Wen X Bi E Kossenkov AV Wolf AI The transcription factor Foxp1 is a critical negative regulator of the differentiation of follicular helper T cells. Nat Immunol (2014) 15:667–75. 10.1038/ni.2890 24859450 PMC4142638

[171] Lee JY Skon CN Lee YJ Oh S Taylor JJ Malhotra D The transcription factor KLF2 restrains CD4(+) T follicular helper cell differentiation. Immunity (2015) 42:252–64. 10.1016/j.immuni.2015.01.013 25692701 PMC4409658

[172] Johnston RJ Choi YS Diamond JA Yang JA Crotty S. STAT5 is a potent negative regulator of TFH cell differentiation. J Exp Med (2012) 209:243–50. 10.1084/jem.20111174 22271576 PMC3281266

[173] Park HJ Kim DH Choi JY Kim WJ Kim JY Senejani AG PPARgamma negatively regulates T cell activation to prevent follicular helper T cells and germinal center formation. PLoS One (2014) 9:e99127 10.1371/journal.pone.0099127 24921943 PMC4055678

[174] Vanderleyden I Linterman MA Smith KG. Regulatory T cells and control of the germinal centre response. Arthritis Res Ther (2014) 16:471. 10.1186/s13075-014-0471-7 25606598 PMC4289055

[175] Wollenberg I Agua-Doce A Hernandez A Almeida C Oliveira VG Faro J Regulation of the germinal center reaction by Foxp3+ follicular regulatory T cells. J Immunol (2011) 187:4553–60. 10.4049/jimmunol.1101328 21984700

[176] Zhu Y Zou L Liu YC. T follicular helper cells, T follicular regulatory cells and autoimmunity. Int Immunol (2016) 28:173–9. 10.1093/intimm/dxv079 26714592 PMC4889881

[177] Dhaeze T Stinissen P Liston A Hellings N. Humoral autoimmunity: a failure of regulatory T cells? Autoimmun Rev (2015) 14:735–41. 10.1016/j.autrev.2015.04.006 25913138

[178] Sage PT Sharpe AH. T follicular regulatory cells in the regulation of B cell responses. Trends Immunol (2015) 36:410–8. 10.1016/j.it.2015.05.005 26091728 PMC4508020

[179] Xu L Huang Q Wang H Hao Y Bai Q Hu J The kinase mTORC1 promotes the generation and suppressive function of follicular regulatory T cells. Immunity (2017) 47:538–551.e5. 10.1016/j.immuni.2017.08.011 28930662

[180] Chang JH Hu H Jin J Puebla-Osorio N Xiao Y Gilbert BE TRAF3 regulates the effector function of regulatory T cells and humoral immune responses. J Exp Med (2014) 211:137–51. 10.1084/jem.20131019 24378539 PMC3892978

[181] Jandl C Liu SM Canete PF Warren J Hughes WE Vogelzang A IL-21 restricts T follicular regulatory T cell proliferation through Bcl-6 mediated inhibition of responsiveness to IL-2. Nat Commun (2017) 8:14647. 10.1038/ncomms14647 28303891 PMC5357862

[182] Maceiras AR Fonseca VR Agua-Doce A Graca L. T follicular regulatory cells in mice and men. Immunology (2017) 152:25–35. 10.1111/imm.12774 28617936 PMC5543727

[183] Sage PT Alvarez D Godec J von Andrian UH Sharpe AH. Circulating T follicular regulatory and helper cells have memory-like properties. J Clin Invest (2014) 124:5191–204. 10.1172/JCI76861 25347469 PMC4348955

[184] Vignali DA Collison LW Workman CJ. How regulatory T cells work. Nat Rev Immunol (2008) 8:523–32. 10.1038/nri2343 18566595 PMC2665249

[185] Laidlaw BJ Lu Y Amezquita RA Weinstein JS Vander Heiden JA Gupta NT Interleukin-10 from CD4(+) follicular regulatory T cells promotes the germinal center response. Sci Immunol (2017) 2:eaan4767 10.1126/sciimmunol.aan4767 29054998 PMC5846620

[186] Hori S Nomura T Sakaguchi S. Control of regulatory T cell development by the transcription factor Foxp3. Science (2003) 299:1057–61. 10.1126/science.1079490 12522256

[187] Mayer CT Floess S Baru AM Lahl K Huehn J Sparwasser T. CD8+ Foxp3+ T cells share developmental and phenotypic features with classical CD4+ Foxp3+ regulatory T cells but lack potent suppressive activity. Eur J Immunol (2011) 41:716–25. 10.1002/eji.201040913 21312192

[188] Joosten SA van Meijgaarden KE Savage ND de Boer T Triebel F van der Wal A Identification of a human CD8+ regulatory T cell subset that mediates suppression through the chemokine CC chemokine ligand 4. Proc Natl Acad Sci U S A (2007) 104:8029–34. 10.1073/pnas.0702257104 17483450 PMC1876566

[189] Kim HJ Verbinnen B Tang X Lu L Cantor H. Inhibition of follicular T-helper cells by CD8(+) regulatory T cells is essential for self tolerance. Nature (2010) 467:328–32. 10.1038/nature09370 20844537 PMC3395240

[190] Kim HJ Wang X Radfar S Sproule TJ Roopenian DC Cantor H. CD8+ T regulatory cells express the Ly49 class I MHC receptor and are defective in autoimmune prone B6-Yaa mice. Proc Natl Acad Sci U S A (2011) 108:2010–5. 10.1073/pnas.1018974108 21233417 PMC3033298

[191] Taghavie-Moghadam PL Waseem TC Hattler J Glenn LM Dobrian AD Kaplan MH STAT4 regulates the CD8(+) regulatory T cell/T follicular helper cell axis and promotes atherogenesis in insulin-resistant Ldlr(-/-) mice. J Immunol (2017) 199:3453–65. 10.4049/jimmunol.1601429 29055004 PMC5675741

[192] Endharti AT Rifa IM Shi Z Fukuoka Y Nakahara Y Kawamoto Y Cutting edge: CD8+CD122+ regulatory T cells produce IL-10 to suppress IFN-gamma production and proliferation of CD8+ T cells. J Immunol (2005) 175:7093–7. 10.4049/jimmunol.175.11.7093 16301610

[193] Holderried TA Kim H-J Lang PA Cantor H CD8^+^ Treg – from mouse to man. Blood (2013) 122:3474.

[194] Miles B Miller SM Folkvord JM Levy DN Rakasz EG Skinner PJ Follicular regulatory CD8 T cells impair the germinal center response in SIV and ex vivo HIV infection. PLoS Pathog (2016) 12:e1005924. 10.1371/journal.ppat.1005924 27716848 PMC5055335

[195] Rampuria P Lang ML. CD1d-dependent expansion of NKT follicular helper cells in vivo and in vitro is a product of cellular proliferation and differentiation. Int Immunol (2015) 27:253–63. 10.1093/intimm/dxv007 25710490 PMC4406267

[196] Chang PP Barral P Fitch J Pratama A Ma CS Kallies A Identification of Bcl-6-dependent follicular helper NKT cells that provide cognate help for B cell responses. Nat Immunol (2011) 13:35–43. 10.1038/ni.2166 22120117

[197] Rampuria P Lang GA Devera TS Gilmore C Ballard JD Lang ML. Coordination between T helper cells, iNKT cells, and their follicular helper subsets in the humoral immune response against Clostridium difficile toxin B. J Leukoc Biol (2017) 101:567–76. 10.1189/jlb.4A0616-271R 27566831 PMC5235901

[198] Lang ML. The influence of invariant natural killer T cells on humoral immunity to T-dependent and -independent antigens. Front Immunol (2018) 9:305. 10.3389/fimmu.2018.00305 29520280 PMC5827355

[199] Tonti E Fedeli M Napolitano A Iannacone M von Andrian UH Guidotti LG Follicular helper NKT cells induce limited B cell responses and germinal center formation in the absence of CD4(+) T cell help. J Immunol (2012) 188:3217–22. 10.4049/jimmunol.1103501 22379027 PMC3559029

[200] Fukazawa Y Lum R Okoye AA Park H Matsuda K Bae JY B cell follicle sanctuary permits persistent productive simian immunodeficiency virus infection in elite controllers. Nat Med (2015) 21:132–9. 10.1038/nm.3781 25599132 PMC4320022

[201] Perdomo-Celis F Taborda NA Rugeles MT Follicular CD8(+) T cells: origin, function and importance during HIV infection. Front Immunol (2017) 8:1241 10.3389/fimmu.2017.01241 29085360 PMC5649150

[202] Quigley MF Gonzalez VD Granath A Andersson J Sandberg JK. CXCR5+ CCR7- CD8 T cells are early effector memory cells that infiltrate tonsil B cell follicles. Eur J Immunol (2007) 37:3352–62. 10.1002/eji.200636746 18000950

[203] He R Hou S Liu C Zhang A Bai Q Han M Follicular CXCR5- expressing CD8(+) T cells curtail chronic viral infection. Nature (2016) 537:412–28. 10.1038/nature19317 27501245

[204] Im SJ Hashimoto M Gerner MY Lee J Kissick HT Burger MC Defining CD8+ T cells that provide the proliferative burst after PD-1 therapy. Nature (2016) 537:417–21. 10.1038/nature19330 27501248 PMC5297183

[205] Leong YA Chen Y Ong HS Wu D Man K Deleage C CXCR5(+) follicular cytotoxic T cells control viral infection in B cell follicles. Nat Immunol (2016) 17:1187–96. 10.1038/ni.3543 27487330

[206] Li S Folkvord JM Rakasz EG Abdelaal HM Wagstaff RK Kovacs KJ Simian immunodeficiency virus-producing cells in follicles are partially suppressed by CD8+ cells in vivo. J Virol (2016) 90:11168–80. 10.1128/JVI.01332-16 27707919 PMC5126374

[207] Petrovas C Ferrando-Martinez S Gerner MY Casazza JP Pegu A Deleage C Follicular CD8 T cells accumulate in HIV infection and can kill infected cells in vitro via bispecific antibodies. Sci Transl Med (2017) 9:eaag2285. 10.1126/scitranslmed.aag2285 28100833 PMC5497679

[208] Ferrando-Martinez S Moysi E Pegu A Andrews S Nganou Makamdop K Ambrozak D Accumulation of follicular CD8+ T cells in pathogenic SIV infection. J Clin Invest (2018) 128:2089–103. 10.1172/JCI96207 29664020 PMC5919804

[209] Rahman MA McKinnon KM Karpova TS Ball DA Venzon DJ Fan W Associations of simian immunodeficiency virus (SIV)-specific follicular CD8^+^ T cells with other follicular T cells suggest complex contributions to SIV viremia control. J Immunol (2018) 200:2714. 10.4049/jimmunol.1701403 29507105 PMC5893362

[210] Curran MA Montalvo W Yagita H Allison JP. PD-1 and CTLA-4 combination blockade expands infiltrating T cells and reduces regulatory T and myeloid cells within B16 melanoma tumors. Proc Natl Acad Sci U S A (2010) 107:4275–80. 10.1073/pnas.0915174107 20160101 PMC2840093

[211] Jin Y Lang C Tang J Geng J Song HK Sun Z CXCR5(+)CD8(+) T cells could induce the death of tumor cells in HBV-related hepatocellular carcinoma. Int Immunopharmacol (2017) 53:42–8. 10.1016/j.intimp.2017.10.009 29032029

[212] Xu H Liu J Cui X Zuo Y Zhang Z Li Y Increased frequency of circulating follicular helper T cells in lupus patients is associated with autoantibody production in a CD40L-dependent manner. Cell Immunol (2015) 295:46–51. 10.1016/j.cellimm.2015.01.014 25748125

[213] Kawamoto M Harigai M Hara M Kawaguchi Y Tezuka K Tanaka M Expression and function of inducible co-stimulator in patients with systemic lupus erythematosus: possible involvement in excessive interferon-gamma and anti-double-stranded DNA antibody production. Arthritis Res Ther (2006) 8:R62. 10.1186/ar1928 16563187 PMC1526621

[214] Wang L Zhao P Ma L Shan Y Jiang Z Wang J Increased interleukin 21 and follicular helper T-like cells and reduced interleukin 10+ B cells in patients with new-onset systemic lupus erythematosus. J Rheumatol (2014) 41:1781–92. 10.3899/jrheum.131025 25028374

[215] Terrier B Costedoat-Chalumeau N Garrido M Geri G Rosenzwajg M Musset L Interleukin 21 correlates with T cell and B cell subset alterations in systemic lupus erythematosus. J Rheumatol (2012) 39:1819–28. 10.3899/jrheum.120468 22859347

[216] Lan Y Luo B Wang JL Jiang YW Wei YS. The association of interleukin-21 polymorphisms with interleukin-21 serum levels and risk of systemic lupus erythematosus. Gene (2014) 538:94–8. 10.1016/j.gene.2014.01.012 24434811

[217] Wong CK Wong PT Tam LS Li EK Chen DP Lam CW. Elevated production of B cell chemokine CXCL13 is correlated with systemic lupus erythematosus disease activity. J Clin Immunol (2010) 30:45–52. 10.1007/s10875-009-9325-5 19774453

[218] Lee J Shin EK Lee SY Her YM Park MK Kwok SK Oestrogen up-regulates interleukin-21 production by CD4(+) T lymphocytes in patients with systemic lupus erythematosus. Immunology (2014) 142:573–80. 10.1111/imm.12263 24495300 PMC4107667

[219] Nakou M Papadimitraki ED Fanouriakis A Bertsias GK Choulaki C Goulidaki N Interleukin-21 is increased in active systemic lupus erythematosus patients and contributes to the generation of plasma B cells. Clin Exp Rheumatol (2013) 31:172–9. 23137515

[220] Liarski VM Kaverina N Chang A Brandt D Yanez D Talasnik L Cell distance mapping identifies functional T follicular helper cells in inflamed human renal tissue. Sci Transl Med (2014) 6:230ra46. 10.1126/scitranslmed.3008146 24695686 PMC4129446

[221] Chang A Henderson SG Brandt D Liu N Guttikonda R Hsieh C In situ B cell-mediated immune responses and tubulointerstitial inflammation in human lupus nephritis. J Immunol (2011) 186:1849–60. 10.4049/jimmunol.1001983 21187439 PMC3124090

[222] Vinuesa CG Cook MC Angelucci C Athanasopoulos V Rui L Hill KM A RING-type ubiquitin ligase family member required to repress follicular helper T cells and autoimmunity. Nature (2005) 435:452–8. 10.1038/nature03555 15917799

[223] Linterman MA Rigby RJ Wong RK Yu D Brink R Cannons JL Follicular helper T cells are required for systemic autoimmunity. J Exp Med (2009) 206:561–76. 10.1084/jem.20081886 19221396 PMC2699132

[224] Bubier JA Sproule TJ Foreman O Spolski R Shaffer DJ Morse HC III A critical role for IL-21 receptor signaling in the pathogenesis of systemic lupus erythematosus in BXSB-Yaa mice. Proc Natl Acad Sci U S A (2009) 106:1518–23. 10.1073/pnas.0807309106 19164519 PMC2635818

[225] Mandik-Nayak L Seo SJ Sokol C Potts KM Bui A Erikson J. MRL-lpr/lpr mice exhibit a defect in maintaining developmental arrest and follicular exclusion of anti-double-stranded DNA B cells. J Exp Med (1999) 189:1799–814. 10.1084/jem.189.11.1799 10359584 PMC2193088

[226] Odegard JM Marks BR DiPlacido LD Poholek AC Kono DH Dong C ICOS-dependent extrafollicular helper T cells elicit IgG production via IL-21 in systemic autoimmunity. J Exp Med (2008) 205:2873–86. 10.1084/jem.20080840 18981236 PMC2585848

[227] Simpson N Gatenby PA Wilson A Malik S Fulcher DA Tangye SG Expansion of circulating T cells resembling follicular helper T cells is a fixed phenotype that identifies a subset of severe systemic lupus erythematosus. Arthritis Rheum (2010) 62:234–44. 10.1002/art.25032 20039395

[228] Bohnhorst JO Bjorgan MB Thoen JE Jonsson R Natvig JB Thompson KM Abnormal B cell differentiation in primary Sjogren’s syndrome results in a depressed percentage of circulating memory B cells and elevated levels of soluble CD27 that correlate with serum IgG concentration. Clin Immunol (2002) 103:79–88. 10.1006/clim.2002.5199 11987988

[229] Szabo K Papp G Barath S Gyimesi E Szanto A Zeher M Follicular helper T cells may play an important role in the severity of primary Sjögren’s syndrome. Clin Immunol (2013) 147:95–104. 10.1016/j.clim.2013.02.024 23578551

[230] Bombardieri M Barone F Humby F Kelly S McGurk M Morgan P Activation-induced cytidine deaminase expression in follicular dendritic cell networks and interfollicular large B cells supports functionality of ectopic lymphoid neogenesis in autoimmune sialoadenitis and MALT lymphoma in Sjogren’s syndrome. J Immunol (2007) 179:4929–38. 10.4049/jimmunol.179.7.4929 17878393

[231] Sidiropoulos PI Boumpas DT. Lessons learned from anti-CD40L treatment in systemic lupus erythematosus patients. Lupus (2004) 13:391–7. 10.1191/0961203304lu1032oa 15230298

[232] Pertovaara M Silvennoinen O Isomaki P Cytokine-induced STAT1 activation is increased in patients with primary Sjogren’s syndrome. Clin Immunol (2016) 165:60–7. 10.1016/j.clim.2016.03.010 26995659

[233] Linsley PS Nadler SG. The clinical utility of inhibiting CD28-mediated costimulation. Immunol Rev (2009) 229:307–21. 10.1111/j.1600-065X.2009.00780.x 19426230

[234] Patakas A Ji RR Weir W Connolly SE Benson RA Nadler SG Abatacept inhibition of T cell priming in mice by induction of a unique transcriptional profile that reduces their ability to activate antigen-presenting cells. Arthritis Rheumatol (2016) 68:627–38. 10.1002/art.39470 26473409

[235] Verstappen GM Meiners PM Corneth OBJ Visser A Arends S Abdulahad WH Attenuation of follicular helper T cell-dependent B cell hyperactivity by abatacept treatment in primary Sjogren’s syndrome. Arthritis Rheumatol (2017) 69:1850–61. 10.1002/art.40165 28564491

[236] Wang L Zhang Y He M. Clinical predictors for the prognosis of myasthenia gravis. BMC Neurol (2017) 17:77. 10.1186/s12883-017-0857-7 28420327 PMC5395963

[237] Luo C Li Y Liu W Feng H Wang H Huang X Expansion of circulating counterparts of follicular helper T cells in patients with myasthenia gravis. J Neuroimmunol (2013) 256:55–61. 10.1016/j.jneuroim.2012.12.001 23305745

[238] Le Coz C Joublin A Pasquali JL Korganow AS Dumortier H Monneaux F. Circulating TFH subset distribution is strongly affected in lupus patients with an active disease. PLoS One (2013) 8:e75319. 10.1371/journal.pone.0075319 24069401 PMC3777901

[239] Romme Christensen J Bornsen L Ratzer R Piehl F Khademi M Olsson T Systemic inflammation in progressive multiple sclerosis involves follicular T-helper, Th17- and activated B-cells and correlates with progression. PLoS One (2013) 8:e57820. 10.1371/journal.pone.0057820 23469245 PMC3585852

[240] Liu R Wu Q Su D Che N Chen H Geng L A regulatory effect of IL-21 on T follicular helper-like cell and B cell in rheumatoid arthritis. Arthritis Res Ther (2012) 14:R255. 10.1186/ar4100 23176102 PMC3674600

[241] An LM Li J Ji LL Li GT Zhang ZL. [Detection of peripheral follicular helper T cells in rheumatoid arthritis]. Beijing Da Xue Xue Bao Yi Xue Ban (2016) 48:951–7. 10.3969/j.issn.1671-167X.2016.06.006 27987496

[242] Rao DA Gurish MF Marshall JL Slowikowski K Fonseka CY Liu Y Pathologically expanded peripheral T helper cell subset drives B cells in rheumatoid arthritis. Nature (2017) 542:110–4. 10.1038/nature20810 28150777 PMC5349321

[243] Moschovakis GL Bubke A Friedrichsen M Falk CS Feederle R Förster R T cell specific Cxcr5 deficiency prevents rheumatoid arthritis. Sci Rep (2017) 7:8933 10.1038/s41598-017-08935-6 28827539 PMC5567121

[244] Kenefeck R Wang CJ Kapadi T Wardzinski L Attridge K Clough LE Follicular helper T cell signature in type 1 diabetes. J Clin Invest (2015) 125:292–303. 10.1172/JCI76238 25485678 PMC4382272

[245] Xu X Shi Y Cai Y Zhang Q Yang F Chen H Inhibition of increased circulating Tfh cell by anti-CD20 monoclonal antibody in patients with type 1 diabetes. PLoS One (2013) 8:e79858. 10.1371/journal.pone.0079858 24278195 PMC3835920

[246] Ferreira RC Simons HZ Thompson WS Cutler AJ Dopico XC Smyth DJ IL-21 production by CD4(+) effector T cells and frequency of circulating follicular helper T cells are increased in type 1 diabetes patients. Diabetologia (2015) 58:781–90. 10.1007/s00125-015-3509-8 25652388 PMC4351433

[247] Viisanen T Ihantola E-L Näntö-Salonen K Hyöty H Nurminen N Selvenius J Circulating CXCR5+PD-1+ICOS+ follicular T helper cells are increased close to the diagnosis of type 1 diabetes in children with multiple autoantibodies. Diabetes (2017) 66:437–47. 10.2337/db16-0714 28108610

[248] Arif S Leete P Nguyen V Marks K Nor NM Estorninho M Blood and islet phenotypes indicate immunological heterogeneity in type 1 diabetes. Diabetes (2014) 63:3835–45. 10.2337/db14-0365 24939426 PMC4207393

[249] Zhou J Wang Y He Y Gao Y Wan R Cai M Non-obese type 2 diabetes patients present intestinal B cell dysregulations associated with hyperactive intestinal Tfh cells. Mol Immunol (2018) 97:27–32. 10.1016/j.molimm.2018.03.008 29550578

[250] Gaddis DE Padgett LE Wu R McSkimming C Romines V Taylor AM Apolipoprotein AI prevents regulatory to follicular helper T cell switching during atherosclerosis. Nat Commun (2018) 9:1095. 10.1038/s41467-018-03493-5 29545616 PMC5854619

[251] Clement M Guedj K Andreata F Morvan M Bey L Khallou-Laschet J Control of the T follicular helper-germinal center B-cell axis by CD8(+) regulatory T cells limits atherosclerosis and tertiary lymphoid organ development. Circulation (2015) 131:560–70. 10.1161/CIRCULATIONAHA.114.010988 25552357

[252] Kamekura R Shigehara K Miyajima S Jitsukawa S Kawata K Yamashita K Alteration of circulating type 2 follicular helper T cells and regulatory B cells underlies the comorbid association of allergic rhinitis with bronchial asthma. Clin Immunol (2015) 158:204–11. 10.1016/j.clim.2015.02.016 25829231

[253] Jin J Kobayashi T Bachman KA Kita H T follicular helper (Tfh) cells are activated by natural exposure to pollens during the Ragweed Hay fever season. J Allergy Clin Immunol (2018) 141:AB176 10.1016/j.jaci.2017.12.561

[254] Dolence JJ Kobayashi T Iijima K Krempski J Drake LY Dent AL Airway exposure initiates peanut allergy by involving the IL-1 pathway and T follicular helper cells in mice. J Allergy Clin Immunol (2017). 10.1016/j.jaci.2017.11.020 29247716 PMC6002896

[255] Akdis CA Akdis M Bieber T Bindslev-Jensen C Boguniewicz M Eigenmann P Diagnosis and treatment of atopic dermatitis in children and adults: European Academy of Allergology and Clinical Immunology/American Academy of Allergy, Asthma and Immunology/PRACTALL Consensus Report. J Allergy Clin Immunol (2006) 118:152–69. 10.1016/j.jaci.2006.03.045 16815151

[256] Eyerich K Novak N. Immunology of atopic eczema: overcoming the Th1/Th2 paradigm. Allergy (2013) 68:974–82. 10.1111/all.12184 23889510

[257] Werfel T Allam JP Biedermann T Eyerich K Gilles S Guttman-Yassky E Cellular and molecular immunologic mechanisms in patients with atopic dermatitis. J Allergy Clin Immunol (2016) 138:336–49. 10.1016/j.jaci.2016.06.010 27497276

[258] Eyerich K Huss-Marp J Darsow U Wollenberg A Foerster S Ring J Pollen grains induce a rapid and biphasic eczematous immune response in atopic eczema patients. Int Arch Allergy Immunol (2008) 145:213–23. 10.1159/000109290 17914273

[259] Szabó K Gáspár K Dajnoki Z Papp G Fábos B Szegedi A Expansion of circulating follicular T helper cells associates with disease severity in childhood atopic dermatitis. Immunol Lett (2017) 189:101–8. 10.1016/j.imlet.2017.04.010 28431963

[260] Czarnowicki T Esaki H Gonzalez J Renert-Yuval Y Brunner P Oliva M Alterations in B-cell subsets in pediatric patients with early atopic dermatitis. J Allergy Clin Immunol (2017) 140:134–44.e9. 10.1016/j.jaci.2016.09.060 27965110

[261] Ballesteros-Tato A Randall TD Lund FE Spolski R Leonard WJ León B. T follicular helper cell plasticity shapes pathogenic T helper 2 cell-mediated immunity to inhaled house dust mite. Immunity (2016) 44:259–73. 10.1016/j.immuni.2015.11.017 26825674 PMC4758890

[262] Ma CS Nichols KE Tangye SG. Regulation of cellular and humoral immune responses by the SLAM and SAP families of molecules. Annu Rev Immunol (2007) 25:337–79. 10.1146/annurev.immunol.25.022106.141651 17201683

[263] Ma CS Hare NJ Nichols KE Dupré L Andolfi G Roncarolo M-G Tangye, Impaired humoral immunity in X-linked lymphoproliferative disease is associated with defective IL-10 production by CD4(+) T cells. J Clin Invest (2005) 115:1049–59. 10.1172/JCI200523139 15761493 PMC1059448

[264] Schmitt N Bustamante J Bourdery L Bentebibel SE Boisson-Dupuis S Hamlin F IL-12 receptor beta1 deficiency alters in vivo T follicular helper cell response in humans. Blood (2013) 121:3375–85. 10.1182/blood-2012-08-448902 23476048 PMC3637013

[265] Lindqvist M van Lunzen J Soghoian DZ Kuhl BD Ranasinghe S Kranias G Expansion of HIV-specific T follicular helper cells in chronic HIV infection. J Clin Invest (2012) 122:3271–80. 10.1172/JCI64314 22922259 PMC3428098

[266] Petrovas C Yamamoto T Gerner MY Boswell KL Wloka K Smith EC CD4 T follicular helper cell dynamics during SIV infection. J Clin Invest (2012) 122:3281–94. 10.1172/JCI63039 22922258 PMC3428091

[267] Perreau M Savoye AL De Crignis E Corpataux JM Cubas R Haddad EK Follicular helper T cells serve as the major CD4 T cell compartment for HIV-1 infection, replication, and production. J Exp Med (2013) 210:143–56. 10.1084/jem.20121932 23254284 PMC3549706

[268] Xu Y Weatherall C Bailey M Alcantara S De Rose R Estaquier J Simian immunodeficiency virus infects follicular helper CD4 T cells in lymphoid tissues during pathogenic infection of pigtail macaques. J Virol (2013) 87:3760–73. 10.1128/JVI.02497-12 23325697 PMC3624224

[269] Xu Y Phetsouphanh C Suzuki K Aggrawal A Graff-Dubois S Roche M HIV-1 and SIV predominantly use CCR5 expressed on a precursor population to establish infection in T follicular helper cells. Front Immunol (2017) 8:376. 10.3389/fimmu.2017.00376 28484447 PMC5399036

[270] Hufert FT van Lunzen J Janossy G Bertram S Schmitz J Haller O Germinal centre CD4+ T cells are an important site of HIV replication in vivo. AIDS (1997) 11:849–57. 10.1097/00002030-199707000-00003 9189209

[271] Kohler SL Pham MN Folkvord JM Arends T Miller SM Miles B Germinal center T follicular helper cells are highly permissive to HIV-1 and alter their phenotype during virus replication. J Immunol (2016) 196:2711–22. 10.4049/jimmunol.1502174 26873986 PMC4779697

[272] Connick E Mattila T Folkvord JM Schlichtemeier R Meditz AL Ray MG CTL fail to accumulate at sites of HIV-1 replication in lymphoid tissue. J Immunol (2007) 178:6975–83. 10.4049/jimmunol.178.11.6975 17513747

[273] Hong JJ Amancha PK Rogers K Ansari AA Villinger F. Spatial alterations between CD4(+) T follicular helper, B, and CD8(+) T cells during simian immunodeficiency virus infection: T/B cell homeostasis, activation, and potential mechanism for viral escape. J Immunol (2012) 188:3247–56. 10.4049/jimmunol.1103138 22387550 PMC3311732

[274] Lu J Lv Y Lv Z Xu Y Huang Y Cui M Expansion of circulating T follicular helper cells is associated with disease progression in HIV-infected individuals. J Infect Public Health (2018). 10.1016/j.jiph.2018.01.005 29409739

[275] De Milito A Nilsson A Titanji K Thorstensson R Reizenstein E Narita M Mechanisms of hypergammaglobulinemia and impaired antigen-specific humoral immunity in HIV-1 infection. Blood (2004) 103:2180–6. 10.1182/blood-2003-07-2375 14604962

[276] Hart M Steel A Clark SA Moyle G Nelson M Henderson DC Loss of discrete memory B cell subsets is associated with impaired immunization responses in HIV-1 infection and may be a risk factor for invasive pneumococcal disease. J Immunol (2007) 178:8212–20. 10.4049/jimmunol.178.12.8212 17548660

[277] Cubas RA Mudd JC Savoye A-L Perreau M van Grevenynghe J Metcalf T Inadequate T follicular cell help impairs B cell immunity during HIV infection. Nat Med (2013) 19:494–9. 10.1038/nm.3109 23475201 PMC3843317

[278] Moir S Fauci AS B cells in HIV infection and disease. Nat Rev Immunol (2009) 9:235–45. 10.1038/nri2524 19319142 PMC2779527

[279] Fahey LM Wilson EB Elsaesser H Fistonich CD McGavern DB Brooks DG. Viral persistence redirects CD4 T cell differentiation toward T follicular helper cells. J Exp Med (2011) 208:987–99. 10.1084/jem.20101773 21536743 PMC3092345

[280] Harker JA Lewis GM Mack L Zuniga EI. Late interleukin-6 escalates T follicular helper cell responses and controls a chronic viral infection. Science (2011) 334:825–9. 10.1126/science.1208421 21960530 PMC3388900

[281] Herati RS Muselman A Vella L Bengsch B Parkhouse K Del Alcazar D Successive annual influenza vaccination induces a recurrent oligoclonotypic memory response in circulating T follicular helper cells. Sci Immunol (2017) 2:eaag2152. 10.1126/sciimmunol.aag2152 28620653 PMC5469419

[282] Koutsakos M Wheatley AK Loh L Clemens EB Sant S Nussing S Circulating TFH cells, serological memory, and tissue compartmentalization shape human influenza-specific B cell immunity. Sci Transl Med (2018) 10: eaan8405. 10.1126/scitranslmed.aan8405 29444980

[283] Wang R Xie R Song Z. Circulating regulatory Tfh cells are enriched in patients with chronic hepatitis B infection and induce the differentiation of regulatory B cells. Exp Cell Res (2018) 365:171–6. 10.1016/j.yexcr.2018.02.031 29501568

[284] Rodrigues V Laforge M Campillo-Gimenez L Soundaramourty C Correia-de-Oliveira A Dinis-Oliveira RJ Abortive T follicular helper development is associated with a defective humoral response in *Leishmania infantum*-infected macaques. PLoS Pathog (2014) 10:e1004096. 10.1371/journal.ppat.1004096 24763747 PMC4005728

[285] Haben I Hartmann W Breloer M. Nematode-induced interference with vaccination efficacy targets follicular T helper cell induction and is preserved after termination of infection. PLoS Negl Trop Dis (2014) 8:e3170. 10.1371/journal.pntd.0003170 25255463 PMC4177885

[286] Perez-Mazliah D Nguyen MP Hosking C McLaughlin S Lewis MD Tumwine I Follicular helper T cells are essential for the elimination of *Plasmodium* infection. EBioMedicine (2017) 24:216–30. 10.1016/j.ebiom.2017.08.030 28888925 PMC5652023

[287] de Leval L Rickman DS Thielen C Reynies A Huang YL Delsol G The gene expression profile of nodal peripheral T-cell lymphoma demonstrates a molecular link between angioimmunoblastic T-cell lymphoma (AITL) and follicular helper T (TFH) cells. Blood (2007) 109:4952–63. 10.1182/blood-2006-10-055145 17284527

[288] Grogg KL Attygalle AD Macon WR Remstein ED Kurtin PJ Dogan A. Expression of CXCL13, a chemokine highly upregulated in germinal center T-helper cells, distinguishes angioimmunoblastic T-cell lymphoma from peripheral T-cell lymphoma, unspecified. Mod Pathol (2006) 19:1101–7. 10.1038/modpathol.3800625 16680156

[289] Vinuesa CG Tangye SG Moser B Mackay CR. Follicular B helper T cells in antibody responses and autoimmunity. Nat Rev Immunol (2005) 5:853–65. 10.1038/nri1714 16261173

[290] Cortes JR Ambesi-Impiombato A Couronné L Quinn SA Kim CS da Silva Almeida AC RHOA G17V induces T follicular helper cell specification and promotes lymphomagenesis. Cancer Cell (2018) 33:259–73.e7. 10.1016/j.ccell.2018.01.001 29398449 PMC5811310

[291] Ochando J Braza MS T follicular helper cells: a potential therapeutic target in follicular lymphoma. Oncotarget (2017) 8:112116–31. 10.18632/oncotarget.22788 29340116 PMC5762384

[292] Gu-Trantien C Loi S Garaud S Equeter C Libin M de Wind A CD4+ follicular helper T cell infiltration predicts breast cancer survival. J Clin Invest (2013) 123:2873–92. 10.1172/JCI67428 23778140 PMC3696556

[293] Bindea G Mlecnik B Tosolini M Kirilovsky A Waldner M Obenauf AC Spatiotemporal dynamics of intratumoral immune cells reveal the immune landscape in human cancer. Immunity (2013) 39:782–95. 10.1016/j.immuni.2013.10.003 24138885

[294] Wang Z Wang Z Diao Y Qian X Zhu N Dong W. Circulating follicular helper T cells in Crohn’s disease (CD) and CD-associated colorectal cancer. Tumour Biol (2014) 35:9355–9. 10.1007/s13277-014-2208-2 24943684

[295] Cha Z Zang Y Guo H Rechlic JR Olasnova LM Gu H Association of peripheral CD4+ CXCR5+ T cells with chronic lymphocytic leukemia. Tumour Biol (2013) 34:3579–85. 10.1007/s13277-013-0937-2 23807677

[296] Shi W Li X Cha Z Sun S Wang L Jiao S Dysregulation of circulating follicular helper T cells in nonsmall cell lung cancer. DNA Cell Biol (2014) 33:355–60. 10.1089/dna.2013.2332 24593033

[297] Xiao H Luo G Son H Zhou Y Zheng W. Upregulation of peripheral CD4+CXCR5+ T cells in osteosarcoma. Tumour Biol (2014) 35:5273–9. 10.1007/s13277-014-1686-6 24519063

